# Enhancement of Cognitive Benefits and Anti-Anxiety Effects of *Phytolacca americana* Fruits in a Zebrafish (*Danio rerio*) Model of Scopolamine-Induced Memory Impairment

**DOI:** 10.3390/antiox14010097

**Published:** 2025-01-15

**Authors:** Lucia-Florina Popovici, Ion Brinza, Florentina Gatea, Georgiana Ileana Badea, Emanuel Vamanu, Simona Oancea, Lucian Hritcu

**Affiliations:** 1 Department of Agricultural Sciences and Food Engineering, “Lucian Blaga” University of Sibiu, 7–9 Ion Ratiu Street, 550024 Sibiu, Romania; luciaflorina.popovici@ulbsibiu.ro; 2 Faculty of Sciences, “Lucian Blaga” University of Sibiu, 7–9 Ion Ratiu Street, 550024 Sibiu, Romania; ionbrinza995@gmail.com; 3 Department of Biology, Faculty of Biology, Alexandru Ioan Cuza University of Iasi, 700506 Iasi, Romania; hritcu@uaic.ro; 4 Centre of Bioanalysis, National Institute for Biological Sciences, 296 Spl. Independentei, 060031 Bucharest, Romania; florentina.gatea@incdsb.ro (F.G.); georgiana.badea@incdsb.ro (G.I.B.); 5 Faculty of Biotechnology, University of Agronomic Science and Veterinary Medicine, 59 Marasti blvd, 011464 Bucharest, Romania; emanuel.vamanu@gmail.com

**Keywords:** *Phytolacca americana*, dementia, memory, anxiety, Alzheimer’s disease, zebrafish, scopolamine, ADMET predictions

## Abstract

*Phytolacca americana* fruits exhibit a wide range of biological activities, including antimicrobial, anti-inflammatory, and anticancer properties. This study aims to investigate the phenolic profile of hydroethanolic extracts from both fresh (PEC) and dried (PEU) fruits of *P. americana* using high-performance liquid chromatography (HPLC) and to evaluate their impact on anxiety-like behavior, memory, oxidative stress, and cholinergic status in zebrafish (*Danio rerio*, Tübingen strain) treated with scopolamine (SCO, 100 μM). Acute administration of PEC and PEU (0.1, 0.5, and 1 mg/L) was conducted for one hour per day. In silico analyses were performed to evaluate the pharmacokinetic characteristics of the phenolic compounds discerned in the two extracts, using platforms such as SwissAdme, Molinspiration, ProToX-III, AdmetLab 3.0, PKCSM, and PASS. Anxiety-like behavior and memory performance were assessed through specific behavioral assays, including the novel tank test (NTT), light/dark test (LD), novel approach test (NAT), Y-maze, and novel object recognition (NOR). Subsequently, the activity of acetylcholinesterase (AChE) and the extent of oxidative stress in the zebrafish brain were investigated. Our findings suggest that both PEC and PEU possess anxiolytic effects, alleviating SCO-induced anxiety and enhancing cognitive performance in amnesic zebrafish. Furthermore, these extracts demonstrated the ability to mitigate cholinergic deficits by inhibiting AChE activity and supporting antioxidant defense mechanisms through increased activity of antioxidant enzymes and reduced lipid and protein peroxidation. These results highlight the potential use of *P. americana* fruit extracts in managing anxiety and cognitive impairments related to dementia conditions.

## 1. Introduction

Knowledge embodies the cognitive function of assimilating information and understanding through contemplation, experience, and sensory perception. This cognitive operation encompasses a multitude of complex intellectual faculties, including attention, memory, logic, decision-making, strategic planning, discernment, cognition, language, and visual–spatial processing. Cognitive procedures use pre-existing knowledge while also generating new cognitive constructions. The term “cognitive impairment” denotes a decline in various domains of cognitive functioning. It is not limited to a specific condition or conditions but, rather, may reflect symptomatic indications of an individual’s underlying health condition. This term is also used interchangeably with “cognitive impairment” and can mean either a transient condition or a progressive and lasting one [1]. Dementia is a broad term used to describe a marked decline in cognitive abilities that interferes with daily activities. Alzheimer’s disease (AD) is the most common form of dementia, accounting for over two-thirds of cases diagnosed in the elderly. AD manifests as a neurodegenerative disorder characterized by a gradual onset and progressive decline of cognitive and behavioral functions, including memory, comprehension, language, attention, reasoning, and judgment. While AD does not directly lead to mortality, it significantly increases susceptibility to other complications that can ultimately lead to death [2]. Confirmed diagnostic criteria for AD include the autopsy identification of neurofibrillary tangles (NFTs) composed of hyperphosphorylated tau protein and toxic oligomeric β-amyloid (Aβ) deposits in the brain [3].

Several studies have validated the presence of cholinergic insufficiency in individuals diagnosed with AD, demonstrating notable deficiencies in choline acetyltransferase (ChAT), the enzyme responsible for the synthesis of acetylcholine (ACh), as well as diminished choline uptake, reduced ACh secretion, and depletion of cholinergic neurons. Given the critical function of ACh in cognitive processes such as learning and memory, damage to cholinergic neurons and impairment of cholinergic neurotransmission contribute significantly to the cognitive decline seen in AD patients [4]. In addition, numerous studies have demonstrated the significant role of brain oxidative stress in the pathology and progression of AD [5,6]. Oxidative stress is defined as a complex process involving the oxidative degradation of proteins, lipids, and other cellular components. It is caused by an imbalance between the production of reactive free radicals and the ability of the antioxidant system to neutralize them [3]. The antioxidant system includes specific enzymes with antioxidant properties. The main indicators of oxidative damage in AD include increased levels of protein carbonyls and 3-nitrotyrosine (3-NT), which reflect protein oxidation. Elevated levels of protein-bound 4-hydroxynonenal (HNE) and an increase in isoprostanes or neuroprostanes, markers of lipid peroxidation, are also observed. All these factors contribute to neuronal dysfunction and disease progression.

Scopolamine (SCO) is known as a muscarinic ACh receptor (mAChR) antagonist and is recognized for its ability to affect learning and memory functions. Its effects on animals and humans are like those seen in AD patients [7]. Therefore, SCO has become a valuable tool in dementia research, being used to create experimental models that enable the evaluation of potential therapies and drug candidates. An important aspect of the action of SCO is the increase in the levels of acetylcholinesterase (AChE) in the cortex and hippocampus of the brain. This enzyme has a crucial role in the degradation of ACh. Low ACh levels are a hallmark of AD, and increasing AChE by SCO administration may represent a method to modulate this deficit. Because of these characteristics, SCO has been widely used in dementia drug research and evaluation. Experimental models involving SCO allow researchers to study the underlying mechanisms of cognitive decline and to identify compounds that might improve cognitive functions in AD patients [8,9].

The zebrafish (*Danio rerio*) represents an emerging neurophysiological model system showing a significant level of conservation with the human genome. It holds all orthologous genes known to be relevant in the context of AD and can recapitulate the specific features of the pathological processes associated with AD in the human body [10]. The neuroanatomical and neurochemical pathways of the zebrafish brain show profound similarities to those of the human brain. Zebrafish, although different from mammals in the general structure of the central nervous system (CNS), show several remarkable similarities at the nuclear and functional levels. Similarities in the structure of the basal ganglia, striatum, hippocampus, amygdala, hypothalamus, and cerebellum, as well as in neurotransmitter systems, provide compelling evidence of significant evolutionary convergence in brain organization and function [11]. In addition, similarities in physiological, emotional, and social behavioral patterns have been well established between these two species, and their data support the use of the zebrafish model for the induction and study of neurodegenerative diseases [12].

Medicinal flora has been used to improve health and treat many ailments dating back to ancient times. An increase in curiosity toward botanical remedies as a complementary or substitute approach to traditional treatment has been observed globally in recent years. Escalation of use is largely attributed to dissatisfaction with the results of conventional therapies, particularly the increased occurrence of adverse effects and perceived inadequacy of efficacy [13].

Species belonging to the genus *Phytolacca*, which falls under the family Phytolaccaceae, have gained recognition for their use in traditional medicine [14]. Of these, *Phytolacca americana* L. (pokeweed) is widely distributed as an indigenous plant in North America and is recognized as an invasive species in various regions around the globe [15]. *P. americana* is a plant with medicinal properties that are well-received in countries such as Korea, Japan, and China [16]. Traditionally, this plant has been used in the form of non-alcoholic liquid extracts, along with topical solutions such as oils, creams, and ointments. These different remedies are claimed to effectively reduce inflammation and address conditions such as edema, nephritis, and others. The usefulness of botanical extracts spans a wide spectrum, including oral health problems, diabetes, and cancer. Specific formulations can be used to treat fungal infections in animals or combat parasitic diseases. *Phytolacca* stands out as a versatile medicinal plant [17]. *P. americana* seeds are abundant in phenolic compounds and possess remarkable biological characteristics. The seed-derived compound, namely americanin A, has shown anti-cancer properties against human colon cancer, while isoamericanin B and C have been shown to inhibit tyrosinase activity [18,19]. In addition, the chemical composition of representatives of the *Phytolacca* genus mainly comprises triterpenoid saponins 3; phytolaccosides A, B, C, D, and E4; jaligonic acid; esculenic acid; phytolaccagenin 5; flavone; phenolic acid; sterol; and polysaccharide [20,21]. Using these botanical specimens, numerous medical conditions have been addressed, and a variety of pharmacological effects have been documented, including antimicrobial, anti-inflammatory, and anti-cancer properties [22,23]. Furthermore, ethnopharmacological data have brought to light the use of this plant in the treatment of skin lesions [24]. Recent research efforts have increasingly deepened the bioactive properties of *P. americana*, including its diuretic, antibacterial, antiviral, anti-inflammatory, antitumor, and anti-hyperplasia effects on mammary glands [25,26].

While pokeweed possesses varying degrees of toxicity in its entirety, the ingestion of pokeberries tends to be generally less harmful than consuming its roots, stems, or leaves. The latter have significantly higher saponin levels than the berries, particularly phytolaccatoxin and phytolaccagenin [27], which can cause gastrointestinal irritation. This aligns with previous research [28] and new ones [29] which confirmed this, showing relatively few significant adverse effects following berry ingestion. However, Mack [28] also indicated that consuming 10 or more pokeberries could result in more severe symptoms, potentially necessitating medical attention, and this supports a possible dose–response relationship.

Apart from its physiological effects, toxicological evaluations such as in vitro erythrocyte agglutination and leukocyte mitosis have been documented [21,30]. This effect may be attributed to a potential hormesis effect of the compounds present in *P. americana* extracts. Hormesis denotes a biphasic dose–response relationship, where low concentrations of a substance or stress factors produce protective effects, while higher doses may induce toxicity. Neurohormesis, an adaptive hormetic response observed within neurons, slows the progression of neurodegenerative disorders and mitigates damage associated with aging, cerebrovascular accidents, and brain injuries. Furthermore, it modulates anxiety, stress, pain, and seizure intensity, representing a novel strategy for managing neurological conditions. Plant-based medicines and dietary supplements, through the hormetic mechanisms of action of their bioactive constituents, have the potential to prevent or alleviate the pathogenesis of neurodegenerative disorders, including AD [31].

Our study proposes that hydroethanolic extracts prepared from *P. americana* fruits, either fresh or dried, could be promising agents in the treatment of conditions associated with dementia, including AD. The bioactive compounds of the extracts, mainly of polyphenolic structure, could serve as strong candidates for the development of new neuropharmacological therapies due to their ability to effectively intervene in the pathological processes leading to cognitive decline through a mechanism similar to the one discussed here, where it reduced oxidative stress and induced activation of the cholinergic system in SCO-treated zebrafish. Therefore, *P. americana* extracts deserve more attention in future research, being a potential innovator in the fight against dementia.

## 2. Materials and Methods

### 2.1. Plant Material

Ripe and undamaged *P. americana* fruits were harvested in November 2023 from uncultivated vegetation of the “Anastasie Fatu” Botanical Garden of Iasi, Romania (47°11′12.5″ N 27°33′14.3″ E). The material was authenticated by Lecturer Biologist Dr. Mihai Craciunas and Principal Biologist Dr. Oprea Adrian. A voucher specimen of this plant was deposited at the Faculty of Sciences Herbarium (No. HFS-231005) of the “Lucian Blaga” University of Sibiu, Romania. The fruits were stored at −20 °C until processing and analysis.

### 2.2. Preparation of Extracts

The fruits of *P. americana* were divided into two groups: one part was kept raw (PEC) followed by blending using the apparatus GRINDOMIX GM 200 (Retsch, Haan, Germany), while the other part was dried (PEU) at 60 °C, using the CI IR D5 Dehydrator (Counter Intelligence, Amstelveen, The Netherlands). The dried samples were ground into powder and sieved to achieve a particle size of 700 μm. Approximately 1 g of fruits (either raw or dried) was extracted with 10 mL 70% aqueous ethanol for 24 h, at r.t. Extracts were concentrated under vacuum at 50 °C using a rotational vacuum concentrator (RVC 2-18 CDplus, Martin Christ GmbH, Osterode am Harz, Germany).

### 2.3. High-Performance Liquid Chromatography (HPLC) Analysis of Phenolic Compounds

The profile and content of phenolic compounds were investigated using a previously published method [32]. The individual phenolic compounds were analyzed using the Shimadzu LC-20AT HPLC system equipped with a quaternary pump, solvent degasser, autosampler, and a photodiode (PDA) detector. The separation of the compounds was performed on a 150 × 4.6 mm, 5 µm Kinetex C18 column (Agilent Technologies, Santa Clara, CA, USA) using mobile phases A (water and phosphoric acid, pH 2.3) and B (acetonitrile) in a gradient mode (between 5 and 90% component B) for 45 min at a temperature of 35 °C, with a flow rate of 0.8 mL/min. The mobile phase was filtered and degassed before a daily run through LLG-Syringe filters, PVDF, 0.20 µm. Standard solutions were prepared for the fifteen investigated phenolic compounds and flavonoids, namely catechin, epicatechin, kaempferol, myricetin, apigenin, quercetin, isoquercitrin, naringenin, *p*-coumaric, protocatechuic, chlorogenic, ferulic, caffeic, ellagic, and gallic acids (all Sigma-Aldrich, Merck, Darmstadt, Germany). The solutions were prepared by dissolving the standards in HPLC-grade methanol (Chromasolv) to produce stock solutions of 1 mg/mL, which were then used to prepare solutions for the standard plots. Following the analysis of the individual phenolic standards UV-Vis spectra, four wavelengths were chosen for identification in this investigation, namely 280 nm (gallic acid, catechin, epicatechin, ellagic acid, apigenin), 260 nm (protocatechuic acid), 320 nm (caffeic, chlorogenic, *p*-coumaric, ferulic acids, naringenin), and 360 nm (myricetin, kaempferol, quercetin, isoquercitrin).

### 2.4. Evaluation of the Physicochemical Properties of Compounds

The evaluation of the physicochemical properties of biocompounds is essential in drug discovery and development, influencing pharmacokinetic and pharmacodynamic parameters. These properties, including the ADME profile and toxicity, help determine the efficacy and safety of a therapeutic candidate. In medicinal chemistry, the analysis of physicochemical properties is combined with the assessment of affinity for biological targets, while the concept of “drug-likeness” guides the selection and optimization of compounds based on criteria such as molecular weight and LogP [33].

SMILES (Simplified Molecular Input Line Entry System) is a simplified text format used to represent chemical structures, with advantages such as ease of use and the ability to be automatically interpreted by software. In the computational analysis, canonical SMILES that were retrieved from the PubChem platform (https://pubchem.ncbi.nlm.nih.gov/ (accessed on 22 December 2024)) were used to represent the biocompounds identified in the PEC extract (protocatechuic acid [C_7_H_6_O_4_], catechin [C_15_H_14_O_6_], epicatechin [C_15_H_14_O_6_], isoquercetin [C_21_H_20_O_12_] and naringenin [C_15_H_12_O_5_] and biocompounds identified in the PEU extract (protocatechuic acid [C_7_H_6_O_4_], chlorogenic acid [C_16_H_18_O_9_], ferulic acid [C_10_H_10_O_4_], myricetin [C_15_H_10_O_8_] and naringenin [C_15_H_12_O_5_]) and compare with SCO and GAL, as indicated in Table 1.

### 2.5. Analysis of Biomedical Alert Structures of the Main Biocompounds Present in PEU and PEC Extracts and Their Analogy with Drugs

To assess the potential biomedical alerts of the compounds SCO, Gal, catechin, epicatechin, isoquercetin, naringenin, protocatechuic acid, chlorogenic acid, ferulic acid, and myricetin, we used the SwissADME platform (http://www.swissadme.ch/index.php# (accessed on 22 December 2024)). The method included the identification of interference compounds using PAINS [34] for the detection of “frequent hitters” and the analysis of fragments associated with false positive results [35]. The list of 105 structural fragments with alerts, created by Brenk [36], was applied to detect toxic or unstable fragments. The assessment of “lead similarity” focused on the physicochemical criteria of the drugs. Additionally, the synthetic accessibility score (SA) [37] was estimated based on a fragment analysis, providing clues about potential difficulties in synthesizing the compounds. The concept of “drug similarity” assesses the potential of a molecule to become a viable oral drug by analyzing bioavailability and physicochemical properties. It involves criteria such as Lipinski’s rule [38] (e.g., molecular weight < 500 Da, Log *p* < 5), Weber’s rule [39] (number of rotating bonds), Ghose’s rule [40] (e.g., molecular weight between 160–480 Da), and Egan’s rule [41], which targets liver toxicity. The SwissADME tool helps calculate these parameters, providing an Abbot bioavailability score for a rapid assessment of the oral absorption potential of compounds, facilitating the selection of promising candidates for drug development.

### 2.6. Estimated In Silico Pharmacokinetic Profile of Biocompounds Isolated from PEC and PEU Extracts

In the computational analysis of biocompounds as well as SCO and GAL, which were tested as controls, we used four free pKCSM [42] platforms, ADMETlab 3.0 [43], and ProTox-III [44] to evaluate different pharmaceutical properties, such as water solubility, Caco2 permeability, intestinal absorption, and interactions with P-glycoprotein. It was necessary to standardize the units of measurement and convert the data into binary categories to ensure the comparability of predictions between different sites. Among the parameters analyzed were skin permeability, distribution in the body (VDss), BBB and CNS permeability, interactions with CYP enzymes, total elimination and toxicity (AMES, acute and chronic oral toxicity, hepatotoxicity, neurotoxicity, immunotoxicity, and skin sensitization). These characteristics are essential to assess the biopharmaceutical and toxicological potential of the studied compounds.

### 2.7. Evaluation of Biological Activity and Identification of Protein Targets of Biocompounds Through the PASS and Molinspiration Platforms

The free online tool PASS [45] was used to assess the biological activity and side effects of the compounds studied. PASS prediction analyzes the relationship between the chemical structure of the substance and the biological activity, providing probabilities for various actions, such as dementia treatments, and anti-inflammatory and antioxidant properties. The results were expressed as scores Pa (probability of activity) and Pi (probability of inactivity), where Pa > 0.5 suggests inhibition of that action and provides more accurate predictions of protein targets for the studied compounds. The Molinspiration platform (https://www.molinspiration.com/ (accessed on 22 December 2024)) was also used. Molinspiration Cheminformatics provided the calculation of drug activities for the 21 drug complexes, providing valuable insights into their biological potential. A score greater than 0.00 indicates significant activity, while values between −0.50 and 0.00 suggest moderate activity, and scores below −0.50 indicate a low likelihood of activity [46].

### 2.8. Animals

Herein, we aim to examine the effects of acute administration of an ethanolic extract of *P. americana* fruits on cognitive functions, anxiety levels, and oxidative status in zebrafish. A total of 135 adult zebrafish (Tubingen strain) were used, coming from a short-fin wild type (Pet Product SRL, Bucharest, Romania), aged 4 months and between 3 and 4 cm long at the beginning of the experiment. These fish, in a balanced ratio of 50:50 between the sexes, were purchased from an authorized breeder (European Zebrafish Resource Center at the Institute of Toxicology and Genetics, Germany), and immediately after purchase, they were acclimatized and rigorously monitored for 15 days under laboratory conditions. To ensure optimal conditions, they were kept in two 70-L tanks equipped with air pumps for constant aeration. The dechlorinated water was treated with Tetra AquaSafe solution (Tetra, Germany) and replaced every 24 h to maintain water quality parameters within appropriate ranges (pH between 7 and 7.5, dissolved oxygen concentrations between 8 ± 1 mg/ L, conductivity levels between 1500 and 1600 µS/cm, and an ammonium and nitrite content of around 0.001 mg/L). In addition, the fish were subjected to a natural lighting regime, simulating the circadian rhythm of their species (14 h of light and 10 h of darkness), and were fed seaweed flakes (Norwin, Gadstrup, Denmark) twice a day at regular times (7 am and 4 pm). For 15 min after feeding, the air pumps were turned off to facilitate its consumption by the fish. All these procedures were in accordance with the ethical regulations and were subject to prior approval by the Animal Ethics Committee at the Faculty of Biology, Alexandru Ioan Cuza University of Iasi, Romania (approval number: 1714/06.07.2023). During the experiment, measures were taken to minimize the discomfort of animals, and none of the study subjects suffered death or signs of intoxication during the treatment period. These precautions and conditions were implemented in accordance with the standards of Directive 2010/63/EU, thus guaranteeing the respect and well-being of the beings involved in the study.

### 2.9. Method of Treatment Administration

**Figure 1:**
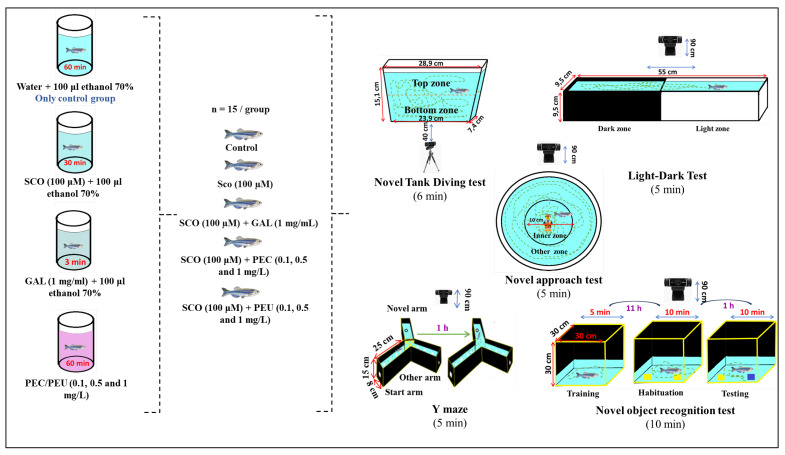
Schematic representation of the experimental design of the study. GAL, galantamine; PEC, ethanolic extracts from fresh fruits of *P. americana*; PEU, ethanolic extracts from dried fruits of *P. americana*; SCO, scopolamine.

After an acclimation period of 15 days, the fish were randomly assigned to 8 experimental groups, each group consisting of 15 animals, in tanks with a capacity of 10 L (30 cm length, 15 cm width, and 15 cm height) and dechlorinated water treated with Tetra AquaSafe, changed daily at 8 am. The water was maintained at a temperature of 26 ± 1 °C, pH between 7 and 7.5, dissolved oxygen concentrations between 8 ± 1 mg/L, conductivity levels between 1500 and 1600 µS/cm, and an ammonium content of around 0.001 mg/L. Thus, one group served as a positive control (Ctr), being treated with 100 μL of 70% ethanol, another group served as a negative control and was treated with SCO (100 μM) + 100 μL 70% ethanol, and one was used as a reference control by being acutely treated with SCO (100 μM) + 100 μL 70% ethanol for 30 min before behavioral tests and with galantamine (GAL, 1 mg/mL) for 3 min before testing. The doses of treatments were chosen based on our previous reports [47,48,49]. The remaining 90 fish exposed to SCO (100 μM) were divided into another 6 groups (Figure 1). Three groups were treated with PEC at a concentration of 0.1, 0.5, and 1 mg/L, and the other three were treated with PEU at a concentration of 0.1, 0.5, and 1 mg/L. Each dose of treatment was dissolved in 100 μL of 70% ethanol. Both PEC and PEU treatments were administered for 15 consecutive days, once a day (8 am), in a 500 mL container for 1 h. The dementia-like state in fish was induced by an acute administration of SCO (100 μM) for 30 min before the novel tank diving test (NTT), the light/dark test (LD), the novel approach test (NAT), and the euthanasia procedure. In the Y maze and novel object recognition test (NOR), administration of SCO (100 μM) occurred 30 min after the training session (Figure 1). Experimental groups were randomly assigned to animals. All animals that were examined were included in the analysis without any attrition or exclusion. All studies were performed according to the planned methodology, and all measured objectives were considered during the analysis. Behavioral assessments were performed by experimentally trained individuals who were unaware of the treatment administered to the zebrafish. Statistical analysis of the data was performed by the experimenters in a blind manner, ensuring that the findings were not influenced by their control. Furthermore, we assert that the use of n = 15 animals per group is appropriate, as determined by InvivoStat and the R-based statistical package.

### 2.10. Behavioral Analysis

To evaluate how acute one-hour administration of PEC and PEU influenced the behavior of the animals included in this study, a zebrafish activity monitoring method was used. Thus, the fish were monitored using a Logitech HD Webcam C922 Pro Stream digital camera (Logitech of Lausanne, Switzerland). Subsequently, the obtained videos were analyzed using ANY maze^®^ software in version 7.44, provided by Stoelting Co. from Wood Dale, Illinois, USA. This methodology allowed us to gain insight into the influence of PEC and PEU on the locomotion, responses, and general behavior of zebrafish while assessing its ability to elicit effects on the CNS and behavior of these organisms in a regulated setting. The examination of the data provided insights into potential changes in environmental exploration, reactions to external stimuli, and other pertinent behaviors.

### 2.11. Novel Tank Diving Test

The novel tank diving test (NTT) is a specific examination used to assess anxiety in zebrafish, as elucidated by Cachat et al. [50]. The equipment used in the current investigation (Figure 1) contained a trapezoidal tank with a capacity of 1.5 L, having dimensions of 15.1 × 23.9 × 7.1 × 28.9 cm, which was divided equally into two sections, namely the top and bottom zones. During the test period, the tank water was changed after each experimental batch and adjusted to 28 °C ± 1 °C using electric heating pads (Seedling Heat Map—HydroFarm). After a 60-min exposure to PEC and PEU treatment, each specimen was individually transferred to a 500 mL beaker containing SCO (100 μM) for 30 min (Figure 1). Immediately after exposure to SCO, each animal was placed in the test apparatus, and locomotor activity was meticulously documented for a duration of 6 min. Latency required for the animal to start the vertical movement of the water column (s), number of entries to the top zone, time spent in the top zone (s), distance traveled in the top zone (m), average entry duration, total distance (m), freezing duration (s) and velocity (m/s) were the key behavioral parameters evaluated in this examination. The test was performed in the time interval 9:00–17:00.

### 2.12. Light/Dark Test

The light/dark test (LDT) is based on the attraction that adult zebrafish have for a dark environment [51]. This analysis of behavior draws attention to the internal conflicts experienced by zebrafish, specifically the conflict between the propensity to stay in areas considered “safe”—in this scenario, the dark environment due to the arrangement of melanophores along the dorsal region that helps reduce bending and light reflection, thus lessening the possibility of being detected by predators—and the inherent desire to explore new surroundings. Despite facing this internal conflict, mature zebrafish show a marked preference for the dark compartment and display light-avoidance behavior [52,53]. The LDT test was conducted in a tank measuring 55 cm long, 9.5 cm high, and 9.5 cm wide, with a white impermeable floor (Figure 1) [53,54,55]. The tank was divided into two perfectly equal sections. Half of the section was covered with white non-reflective waterproof paper, and the second section was covered with black non-reflective waterproof paper. The tank was placed inside a three-sided white enclosure to minimize external visual stimuli. During the test period, the tank arena was filled with 5 cm of water from the fish housing tanks. Water was maintained at 27 °C ± 1 °C using electric heating pads (Seedling Heat Map—HydroFarm) placed under the test tank. During the test period, with utmost care, one fish from a respective group was randomly picked up and gently positioned in the focal point of the light/dark arena. Each fish was placed in the tank after it was aligned parallel to the long axis of the arena, thus preventing any inclination of the fish to the lighted or shaded area. The recording of the fish’s behavior was monitored as soon as the animal was placed in the arena. Immediately after every seventh fish in each group was tested, the LDT was rotated 180° to prevent any possible confusion due to potential resulting biases. Locomotion of zebrafish in the LDT was recorded for 5 min. Dependent variables measured during the test included time spent in the open area (s), time spent in the dark area (s), total distance traveled (m), latency required for fish to start swimming in the open area (s), average speed of swimming (m/s), and preference (%), which was calculated by subtracting the time spent in the open area from the time spent in the dark area.

### 2.13. Novel Approach Test

To examine both locomotion and anxiety-related behavior in zebrafish to novel stimuli, we used the novel approach test (NAT) (Figure 1), as previously detailed by Hamilton [56]. For this, an opaque white plastic cylinder was used, having a diameter of 34 cm and walls with a height of 15 cm, in which water was added from the accommodation environment. Water depth was maintained at 6 cm, and water temperature was adjusted to 27 °C ± 1 °C using electric heating pads (Seedling Heat Map—HydroFarm) placed under the test tank. After each experimental series, the water was changed. In the NAT, the arena was divided into two equal zones: the inner zone and the outer zone (thigmotaxis zone). The central area consisted of a 10 cm diameter circle with the center above the object. The thigmotaxis zone was a circular area located on the wall of the arena, at 4.5 cm from its center, as previously stated by Krook [57]. Fish locomotion was recorded for 5 min. Dependent variables measured during the test included time spent in the inner zone (s), time spent in the outer zone(s), distance traveled (m), immobility time (s), and latency (s). The fish were released into the test tank facing the novel object. The object chosen for this test was a multicolored Lego figurine with a height of 5 cm, following previous analyses by Johnson et al. [58]. The figurine was positioned in the center of the aquarium, and the behavioral test was conducted between 9:00 am and 4:00 pm.

### 2.14. Y-Maze Test

To examine the influence of PEC and PEU on the spatial memory and novelty response of zebrafish, we performed the Y-maze test (Figure 1) based on the experimental procedures developed by Cognato et al. [59]. The Y-maze test was conducted in a tank that was carefully constructed using transparent glass material. The tank was specially designed in a Y-shape, with three arms of equal length. Each arm measured 25 cm long, 8 cm wide, and 15 cm high. These measurements resulted in the formation of an angle of 120 degrees. To increase the efficiency of the video recording, a layer of white plastic was spread over the floor of the tank, while the areas that were not covered were wrapped with black plastic. These sections were then decorated with white visual markers, especially squares, circles, and triangles. In the initial phase of the examination (training), the fish were allowed to investigate only two of the three arms of the maze—the start arm and the familiar arm—while the third arm, symbolizing the unfamiliar (novel) arm, was obstructed. During the testing session, fish were reintroduced to the start arm, with unrestricted entry to all three arms, for 5 min. Immediately after releasing the fish into the start arm, the fish’s behaviors in response to novel stimuli were recorded using a camera mounted above the tank. Subsequently, the video data were analyzed to determine the number of arm entries, the number of line crossings, the percentage of spontaneous alternation (%), turn angle (°), total distance traveled (m), and the time spent in the novel arm (s).

### 2.15. Novel Object Recognition Tests

To assess the impact of acute administration of PEC and PEU on the recognition memory of zebrafish, we implemented the novel object recognition test (NOR) following the protocol established by Stefanello et al. [60]. For NOR (Figure 1), we used a cube-shaped glass tank with dimensions of 30 cm in width, length, and height. The outer walls of the tank were covered with black waterproof paper to reduce the stress on the fish and minimize experimental errors. The test floor was covered with white, non-permeable, non-reflective paper. The tank was placed on a flat surface and filled with water taken from the fish holding tank, the water level reaching 5 cm from the floor. The test lasted four days. During the first three days, each fish was familiarized with the test tank for 5 min twice a day with a 5 h interval between sessions. The training session, conducted 12 h after the last familiarization session, involved the exploration of two identical geometric shapes (yellow cubes with a side of 2.5 cm) for 10 cm. The cubes were placed in two opposite corners of the tank, oriented parallel, with a distance of 10 cm between them.

After the training phase, there was a one-hour retention period during which one of the yellow cubes was replaced by a blue cube. Thus, the yellow cube was considered familiar (OF), and the blue cube was novel (ON). In the subsequent test session, each fish had the opportunity to explore the two cubes for 10 min. Recognition memory was assessed as percentage preference, calculated as the difference in exploration time between ON and OF cubes relative to total exploration time.

### 2.16. Determination of Acetylcholinesterase (AChE) Activity, Antioxidant Enzyme Activity, and Markers of Oxidative Stress

Once the behavioral tests were concluded, all zebrafish involved in this study were euthanized by individually immersing them in a glass container filled with ice-cold water kept at a temperature ranging between 2 and 4 °C. This immersion lasted for 10 min or until no further movements of the opercles or tail were detected [61]. As previously shown [62], this method is recognized for its effectiveness in preventing biochemical and physiological alterations that may otherwise interfere with post-mortem analysis.

Fish brains (∼3 mg) were individually weighed and homogenized in phosphate extraction buffer (0.1 M KH_2_PO_4_, pH 7.4 with 1.15% KCl) at a ratio of 1:10 using a mill ball (Mikro-Dismembrator U; Sartorius, New York, NY, USA) equipped with 3 mm diameter magnetic balls (Sartorius Stedim Biotech GmbH, Göttingen, Germany). The homogenate was centrifuged (15 min at 14,000 rpm) and the supernatant was subsequently used as described in detail previously, for (a) measurement of total protein content [63], (b) determination of the AChE-specific activity [64], (c) determination of the specific activity of catalase (CAT) [56], superoxide dismutase (SOD) [65] and glutathione peroxidase (GPX) [66] enzymes, (d) measurement of total reduced glutathione (GSH) [67] and carbonylated proteins [68] contents, and (e) quantification of malondialdehyde (MDA) level [69].

### 2.17. Statistical Analysis

The statistical analysis was performed using GraphPad Prism 9.5 software (GraphPad Software, Inc., San Diego, CA, USA). All data were expressed as mean ± standard error of the mean (S.E.M). Differences between groups were evaluated using one-way analysis of variance (ANOVA), followed by Tukey’s post-hoc test for multiple comparisons. The level of statistical significance was set at *p* < 0.05. To ensure objectivity, the experimenters conducted data analysis in a blinded manner, without knowledge of group allocation.

## 3. Results

### 3.1. HPLC Analysis of Phenolic Compounds from P. americana Extracts

**Figure 2:**
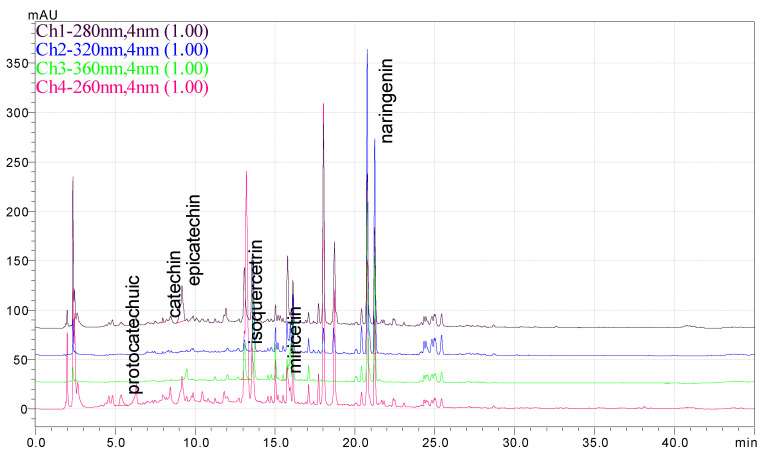
HPLC chromatogram of PEC fruit extract.

**Figure 3:**
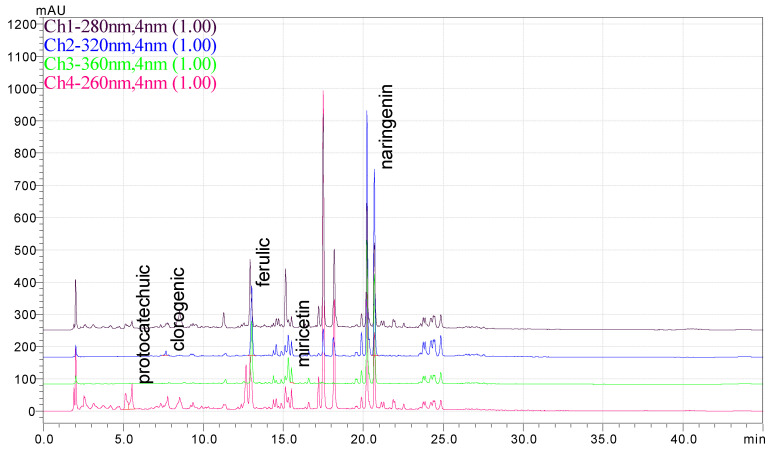
HPLC chromatogram of PEU fruit extract.

The separation of phenolic compounds of PEC and PEU extracts is presented in Figure 2 and Figure 3, while their content is given in Table 2.

The most abundant phenolic compounds of the PEC extract were epicatechin (48.96 ± 1.12 µg/mL), followed by naringenin (21.79 ± 0.15 µg/mL), while in the PEU extract, naringenin (119.07 ± 0.29 µg/mL), ferulic acid (21.79 ± 0.15 µg/mL), and protocatechuic acid (22.93 ± 1.06 µg/mL) were found in high amounts. Protocatechuic acid, chlorogenic acid, and catechin were found in low amounts.

### 3.2. Identification of Biomedical Signaling Structures and the Pharmacokinetic Characteristics of the Main Biocompounds in PEU and PEC

**Figure 4:**
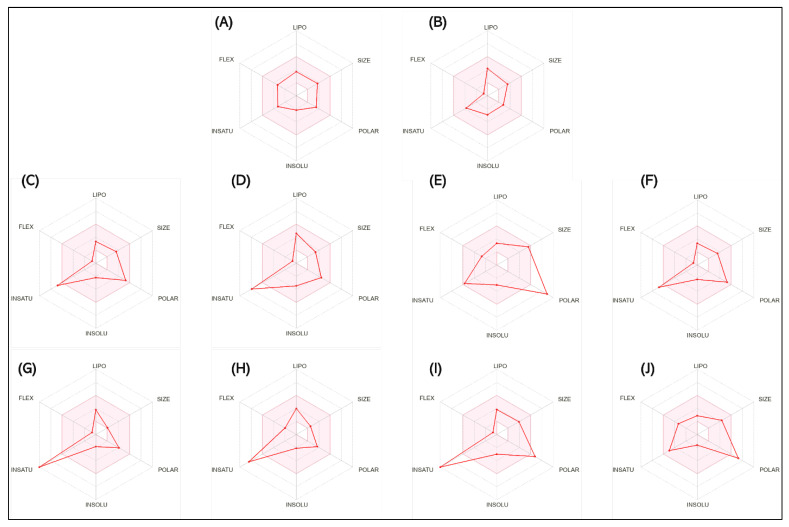
The bioavailability radar delimiting the optimal range for each physicochemical characteristic. In terms of lipophilicity, the pink region is characterized by octanol/water partition coefficient (LogP) values ranging from −0.7 to +5.0. In terms of size, the optimal range is defined by a molecular weight that falls between 150 and 500 g/mol. Polarity is quantified by the total polar surface (TPSA), which ranges from 20 to 130 Å. Solubility is associated with LOgs values that are less than 6. Saturation is represented by the proportion of carbon atoms exhibiting sp3 hybridization, which must exceed 0.25. Flexibility is assessed by a maximum of nine revolving bonds. (**A**) scopolamine (SCO), (**B**) galantamine (GAL), (**C**) epicatechin, (**D**) naringenin, (**E**) isoquercetin, (**F**) catechin, (**G**) protocatechuic acid, (**H**) ferulic acid, (**I**) myricetin, and (**J**) chlorogenic acid are orally soluble.

Figure 4 shows the bioavailability radar of the studied compounds, which highlights six physicochemical characteristics: lipophilicity, size, polarity, solubility, flexibility, and saturation. The obtained data indicate that all 10 analyzed compounds (SCO, GAL, protocatechuic acid, catechin, epicatechin, isoquercetin, naringenin, chlorogenic acid, ferulic acid, and myricetin) are soluble.

According to research conducted by Xiong et al. [70], compounds with pharmaceutical potential must comply with certain specific physicochemical ranges, detailed in Table 3. Thus, their molecular weight must be between 100 and 600 g/mol. The optimal range for the number of hydrogen bond acceptors (nH) is between 0 and 12, while for the number of hydrogen bond donors (nHD), the ideal value is considered between 0 and 6. As for the total polar area (TPSA), it must be between 20 and 130 Å. To refine the selection, the fraction of hybridized carbon atoms sp^3^ (Csp3) must not be less than 0.25, and the number of rotating bonds must not exceed the limit of 9.

The findings on predictive models of biomedical alert structures for SCO, GAL, epicatechin, naringenin, isoquercetin, catechin, protocatechuic acid, ferulic acid, myricetin, and chlorogenic acid are delimited in Table 4. The evaluation indicated that SCO, GAL, epicatechin, naringenin, and catechin meet the five pharmacokinetic criteria, thus qualifying them as promising candidates for pharmaceutical development, while isoquercetin, protocatechuic acid, ferulic acid, myricetin, and chlorogenic acid do not fully comply with these standards and exhibit moderate bioavailability. The Abbot bioavailability score implies a reasonable probability of oral bioavailability for SCO, GAL, epicatechin, naringenin, catechin, protocatechuic acid, ferulic acid, and myricetin as opposed to a diminished probability for isoquercetin and chlorogenic acid. These in silico evaluations provide an initial assessment and require corroboration through empirical laboratory studies to substantiate the drug potential of these compounds.

### 3.3. Evaluation of the Pharmacological Properties

Table 5 presents the data obtained through the online platforms for predicting the pharmacokinetic properties of the compounds analyzed through the pKCSM e values obtained, which illustrate the absorption and permeability parameters for each compound, including intestinal absorption, skin permeability, and interaction with P-glycoprotein transporters (P-gp), providing essential clues on the bioavailability and ability of the compounds to cross biological barriers. As for intestinal absorption, it is evaluated as a percentage of total absorption, with a reference threshold of 30% to classify absorption as low (<30%) or high (>30%). Of the compounds analyzed, GAL, ferulic acid, and naringenin showed the highest values (>90%), suggesting a potentially excellent intestinal absorption. The compounds SCO, catechin, epicatechin, protocatechuic acid, and myricetin recorded intermediate values (65–72%), indicating a moderate-high absorption profile. On the other hand, isoquercetin and chlorogenic acid showed lower absorption, with values of 47.999% and 36.377%, respectively. Permeability through the skin is indicated by logKp values, with a reference of −2.5, below which permeability is considered high. All compounds exhibit log Kp values below this threshold, suggesting high skin permeability. SCO and GAL recorded the lowest values (−4.097 and −3.75), indicating superior permeability. In terms of interaction with P-glycoprotein (P-gp), most compounds, including SCO, epicatechin, naringenin, isoquercetin, catechin, myricetin, and chlorogenic acid, are substrates of P-gp, which can limit their tissue concentrations by active effluster. This characteristic can influence distribution and bioavailability in certain tissues. In contrast, no compound behaved as an inhibitor of P-gp I or II, suggesting a reduced likelihood of drug interactions associated with P-gp inhibition and maintenance of homeostasis in the transport of other P-gp substrates.

Distribution data included volume of apparent distribution at equilibrium (VDss), unbound fraction in plasma, permeability through the blood/brain barrier (BBB), and permeability in the central nervous system (CNS). VDss (log L/kg) provides an estimate of the distribution of compounds in tissues, with values > 0.45 indicating an extensive distribution and values < −0.15 suggesting a distribution limited to the plasma compartment. Most compounds have VDss values above 0.45, suggesting a wide distribution, especially isoquercetin (1.846) and myricetin (1.317); in contrast, naringenin (−0.015), protocatechuic acid (−1.298) and ferulic acid (−1.367) show negative values, suggesting a lower distribution outside the plasma. The unbound fraction (Fu) was evaluated to indicate the proportion of the freely circulating compound available for distribution and biological activity. Compounds with high values of the unbound fraction have a potentially higher bioavailability. Chlorogenic acid and protocatechuic acid have the highest values (0.658 and 0.648), indicating a substantially unbound fraction. In contrast, naringenin exhibits a low unbound fraction (0.064), suggesting strong plasma protein binding.

We also evaluated the BBB permeability, assessed by log BB. According to pkCSM data, a compound can cross the BBB, with values > 0.3 suggesting penetration and values < 0.1 indicating the impossibility of crossing it. No compound exceeds the threshold of 0.3, suggesting limited permeability by the BBB. The GAL has a value relatively close to the threshold (0.081), while the rest of the compounds, including myricetin and isoquercetin, have substantially lower values, suggesting a low probability of penetration. Similarly, we evaluated Log PS to predict the compound’s ability to penetrate the CNS, with values > −2 indicating penetration potential and values < −3 suggesting the inability to cross it. All compounds have values below −3, suggesting reduced permeability in CNS. Naringenin has a relatively higher value (−2.215) but is still below the effective penetration threshold.

**Figure 5:**
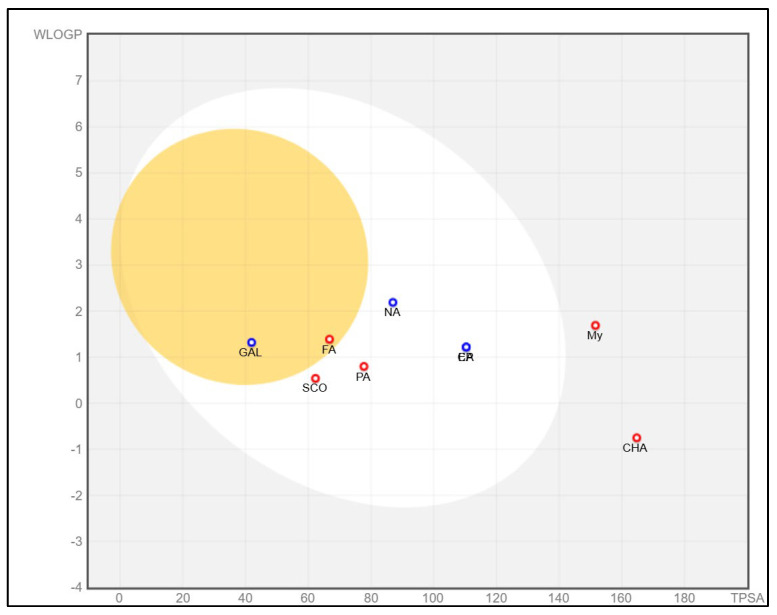
Intuitive assessment of passive gastrointestinal absorption of scopolamine (SCO), galantamine (GAL) compounds, biocompounds identified in PEC such as protocatechuic acid (PA), catechin (CA), epicatechin (EP), isoquercetin (IQ), and naringenin (NA) and biocompounds identified in PEU, such as protocatechuic acid (PA), chlorogenic acid (CHA), ferulic acid (FA), myricetin (MY), and naringenin (NA), as well as their ability to cross the BBB and reach the brain, was achieved by molecular positioning within the WLOGP versus TPSA referential. The classification chart illustrates different aspects of drug absorption and penetration through color coding. The white region indicates a high probability of passive absorption in the gastrointestinal tract, while the yellow region suggests an increased probability of penetration into the brain. These two regions are not mutually exclusive. In addition, the dots are colored blue if they are expected to be actively removed by P-gp (PGP+) and red if they are predicted to be non-substrates of P-gp (PGP-).

At the same time, to identify the ability of at least one of the analyzed compounds in PEC and PEU to cross the BBB and reach the CNS, we used the SwissAdme platform to build a spherical classification diagram. This diagram shows the physicochemical domains associated with BBB permeability and human intestinal absorption, symbolically represented by a hard-boiled egg model (Figure 5). The yolk illustrates the physicochemical space for the very probable BBB permeation, and the white represents the physical/chemical space for the very probable passive gastrointestinal absorption. The gray outer area defines molecules characterized by limited brain absorption and penetration.

The results of the in silico prediction, as shown in Figure 5, indicate that of the biocompounds present in the two extracts of *P. americana*, only the compound ferulic acid (FA), detected in PEU, can cross the BBB (yellow zone), it can also remain in the brain for a long time, not being exposed to rapid expulsion (red dot) compared to GAL, which can easily penetrate the BBB, thus presenting the potential to reach the brain (yellow area), but it will be quickly effluxed from the body as a result of the fact that it can be a substrate for P-gp (blue dot). Regarding the SCO and protocatechuic acid (PA) that have been identified in both PEU and PEC, our prediction indicates that they have a high probability of human passive intestinal absorption (white area) and are not actively eliminated (marked with a red dot). Additionally, a high human passive intestinal absorption (white area) is presented by naringenin (NA), catechin (CA), and epicatechin (EP), but they have an increased potential to be actively expelled via P-gp (blue dot). Chlorogenic acid (CHA) and myricetin (MY), identified in PEU, exhibit potential absorption and a low probability (gray area) of crossing the BBB. In contrast, isoquercetin (IQ) has not been identified on the predictable radar, suggesting its poor absorption. Therefore, results suggest that most compounds exhibit extensive distribution in tissues but limited permeability through the BBB and CNS.

At the same time, as shown in Table 5, we estimated the metabolic interactions of the compounds with the enzymes CYP3A4 and CYP1A2 of the cytochrome P450 (CYP) family, which play an important role in the biotransformation and elimination of active substances in the body [71]. Our data suggest that only SCOP and GAL are identified as substrates of the enzyme CYP3A4, indicating that their metabolism is influenced by its activity. This may suggest a potential for drug interactions, particularly when these compounds are administered concomitantly with CYP3A4 inhibitors or inducers, which could alter the rate of metabolism and, implicitly, therapeutic efficacy and safety. In contrast, naringenin and myricetin are indicated as inhibitors of CYP1A2, suggesting that these compounds may reduce the enzymatic activity of CYP1A2. Inhibition of CYP1A2 can slow down the metabolism of other substances activated by this enzyme, increasing the potential for accumulation and toxicity of those substances [71].

Synchronously, to develop an insight into the rate of elimination of compounds from the body and the mechanisms involved in this process, we analyzed data on compound excretion, including total clearance and possible interaction with the renal transporter OCT2. Our prediction expresses that SCOP and GAL exhibit higher clearance values (1.096 and 0.991), which indicates faster elimination and a shorter half-life, which could lead to a shorter duration of action in the body. In contrast, naringenin (0.06), catechin, and epicatechin (0.183) have lower clearance rates, suggesting slower elimination, which may prolong the time of action. Similarly, only GAL is indicated as a substrate for OCT2, which means that its renal excretion could be more efficient due to the transport facilitated by OCT2. The other compounds are not OCT2 substrates, indicating that their renal elimination is likely limited to glomerular filtration and passive excretion processes [72].

In addition, we predicted the toxicological profile of the compounds identified in PEC and PEU, compared to SCO and GAL (Table 5). Obtained values such as those for GAL (0.458 at normal concentration and 0.625 at 10 μM) and myricetin (0.706 at 10 μM) indicate a moderate potential for hERG channel blockade, suggesting a higher cardiotoxic risk compared to compounds such as chlorogenic acid (0.025 and 0.093), isoquercetin, (0.913), and myricetin (0.839), which have higher DILI (Drug-Induced Liver Injury) values, suggesting a higher risk of causing liver damage, while compounds such as SCOP (0.104) and GAL (0.215) have a lower risk in this regard. Simultaneously, isoquercetin (0.856) and chlorogenic acid (0.386) exhibit higher AMES (mutagenic potential) values, which could indicate a higher probability of producing genetic mutations compared to SCO (0.158), which has a lower risk. In parallel, GAL (0.726) and naringenin (0.591) have higher values of carcinogenicity, which may signal an increased risk of carcinogenicity, while SCO (0.015) and chlorogenic acid (0.225) have much lower values. Similarly, SCO (0.895) and GAL (0.795) have a higher possibility of liver toxicity, while compounds such as myricetin (0.325) have a lower risk. Additionally, SCO (0.753) and GAL (0.744) have higher values, suggesting potential neurotoxicity, while myricetin (0.001) and isoquercetin (0.002) have extremely low values, suggesting a minimal risk in this regard. In terms of hematotoxicity, GAL (0.505) has a moderately high value compared to myricetin (0.015) and chlorogenic acid (0.028), which have much lower values. The genotoxicity data show that epicatechin (0.975) and naringenin (0.978) have high values, suggesting a high risk of genotoxicity, compared to compounds such as chlorogenic acid (0.243), which have a lower risk. Similarly, epicatechin (0.959) and catechin (0.937) are at increased risk of cytotoxicity, suggesting a potential negative effect at the cellular level. However, no compound exhibits immunotoxicity, and most of the compounds identified in PEC and PEU exhibit a lower toxic potential than GAL.

### 3.4. In Silico Prediction of Biological Activity and Potential Protein Targets of Biocompounds Identified in PEC and PEU

**Figure 6:**
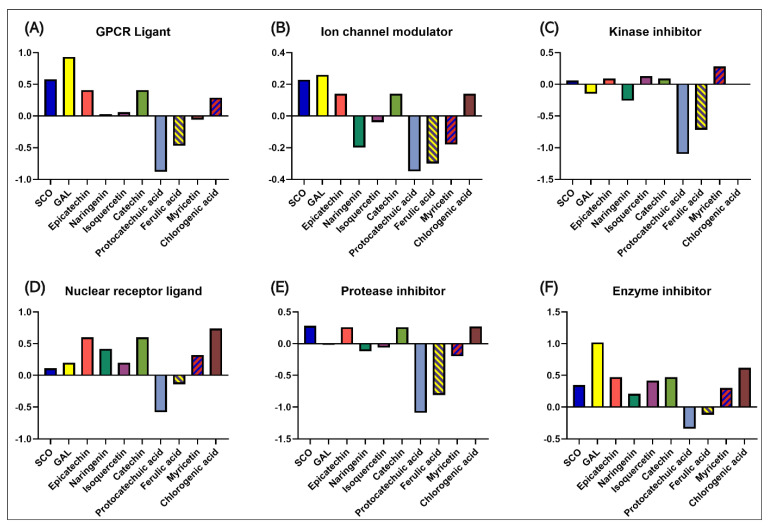
Bioactivity score of SCO, GAL, epicatechin, naringenin, isoquercetin, catechin, protocatechuic acid, ferulic acid, myricetin, and chlorogenic acid, using Molinspiration Cheminformatics software (https://www.molinspiration.com/ (accessed on 22 December 2024)), on (**A**) G protein-coupled receptor ligand (GPCR), (**B**) ion channel modulation, (**C**) kinase inhibition, (**D**) nuclear receptor ligand, (**E**) protease inhibitors, and (**F**) enzyme inhibitors.

Figure 6 shows the bioactivity scores of SCO and GAL, as well as compounds identified in PEC and PEU that were calculated using Molinspiration Cheminformatics (https://www.molinspiration.com/). In the field of G protein-coupled receptors (GPCRs), evaluations carried out by Molinspiration Cheminformatics show that SCO, GAL, epicatechin, naringenin, isoquercetin, catechin, and chlorogenic acid obtained positive scores (exceeding 0.00), indicating an increased ability to influence significant biological activities associated with these receptors. In contrast, protocatechuic acid, ferulic acid, and myricetin showed negative values and most likely did not interact with GPCR receptors (Figure 6A). Regarding ion channel modulation, the peak scores were marked by GAL (0.26) and SCO (0.26), followed by catechin, epicatechin, and chlorogenic acid, while isoquercetin (−0.04), myricetin (−0.18), naringenin (−0.2), ferulic acid (−0.3), and protocatechuic acid (−0.35) received negative scores (Figure 6B). In the context of the potential to inhibit kinase activity, our data suggest that this can only be achieved by SCO, epicatechin, isoquercetin, catechin, and chlorogenic acid, with the other compounds showing scores below 0 (Figure 6C). Concerning the possibility of interaction with the nuclear receptor ligand, negative values were recorded only by protocatechuic acid (−0.58) and ferulic acid (−0.14); in contrast, all the other compounds analyzed, including GAL and, to a lesser extent, SCO, can interact with the nuclear receptor ligand (Figure 6D). SCO (0.28), catechin (0.26), epicatechin (0.26), and chlorogenic acid (0.26) show a high potential to inhibit proteases, followed by GAL (0.01), but protocatechuic acid, isoquercetin, naringenin, ferulic acid, and myricetin marked values below 0 (Figure 6E). Moreover, except for protocatechuic acid and ferulic acid, the other compounds showed the ability to suppress the function of various enzymes (Figure 6F).

**Figure 7:**
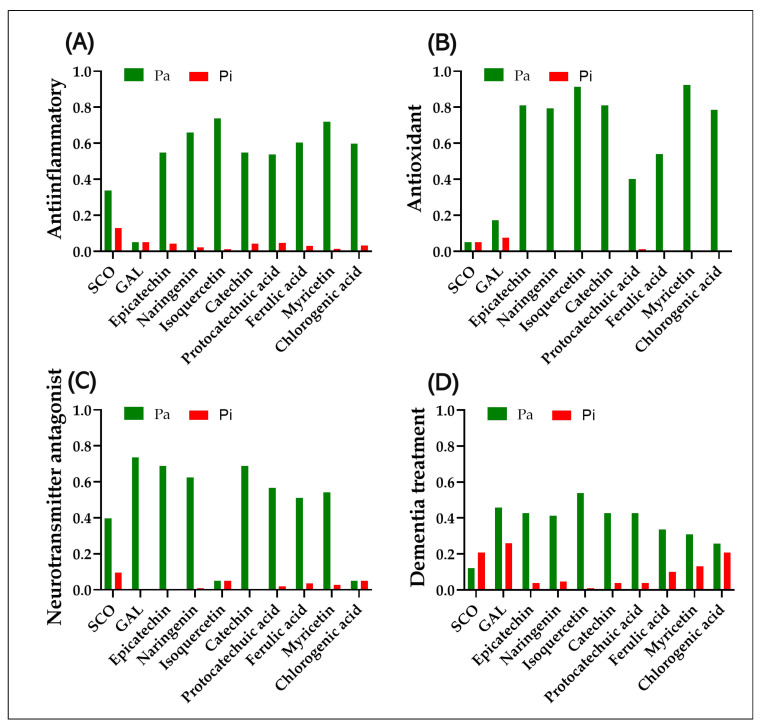
PASS prediction results of SCO, GAL, epicatechin, naringenin, isoquercetin, catechin, protocatechuic acid, ferulic acid, myricetin, and chlorogenic acid on (**A**) anti-inflammatory, (**B**) antioxidant, (**C**) neurotransmitter antagonist, and (**D**) pharmacological potential on dementia. Pa—active; Pi—inactive.

Figure 7 illustrates the results of the PASS predictive analysis, which serves as a valuable tool for assessing the active or inactive potential of a compound within certain biological functionalities using the Pa (active) and Pi (inactive) scores.

The PASS prediction indicated that the most significant anti-inflammatory activity is exhibited by naringenin (Pa of 0.66 and Pi of 0.021), isoquercetin (Pa of 0.739 and Pi of 0.011), ferulic acid (Pa of 0.604 and Pi of 0.031), myricetin (Pa of 0.72 and Pi of 0.013), and chlorogenic acid (Pa of 0.598 and Pi of 0.032), followed by epicatechin (Pa of 0.548 and Pi of 0.044), catechin (Pa of 0.548 and Pi of 0.044), and protocatechuic acid (Pa of 0.538 and Pi of 0.046), while GAL (Pa of 0.05 and Pi of 0.05) and SCO (Pa of 0.338 and Pi of 0.13), have a low potential to manifest anti-inflammatory activity (Figure 7A). Regarding the antioxidant potential, it was noted that all the compounds identified in the two extracts of *P. americana* exhibit a high antioxidant potential. GAL is also represented as a compound with a potential antioxidant effect; in contrast, SCO is not (Figure 7B). Isoquercetin and chlorogenic acid are most likely not neurotransmitter antagonists, as can be seen in Figure 7C, but SCO has a medium potential to be; in contrast, GAL, epicatechin, naringenin, catechin, protocatechuic acid, ferulic acid, and myricetin have the potential to be neurotransmitter antagonists. In addition, according to the PASS prediction, GAL (Pa = 0.458; Pi = 0.26) and isoquercetin (Pa = 0.538; Pi = 0.009) have the highest score of being effective as an anti-dementia drug (Figure 7D), they are closely followed by epicatechin (Pa = 0.426; Pi = 0.038), naringenin (Pa = 0.412; Pi = 0.046), catechin (0.426; Pi = 0.038), protocatechuic acid (Pa = 0.427; Pi = 0.038), and ferulic acid (Pa = 0.336; Pi = 0.101) (Figure 7D). Myricetin (Pa = 0.309; Pi = 0.131) and chlorogenic acid (Pa = 0.258; Pi = 0.209) would most likely be inefficient, and SCO (Pa = 0.121; Pi = 0.208) has a greater potential to induce than serve as an anti-dementia drug (Figure 7D).

### 3.5. Effects of PEC and PEU on Zebrafish Anxiety in the Novel Tank Diving Test

**Figure 8:**
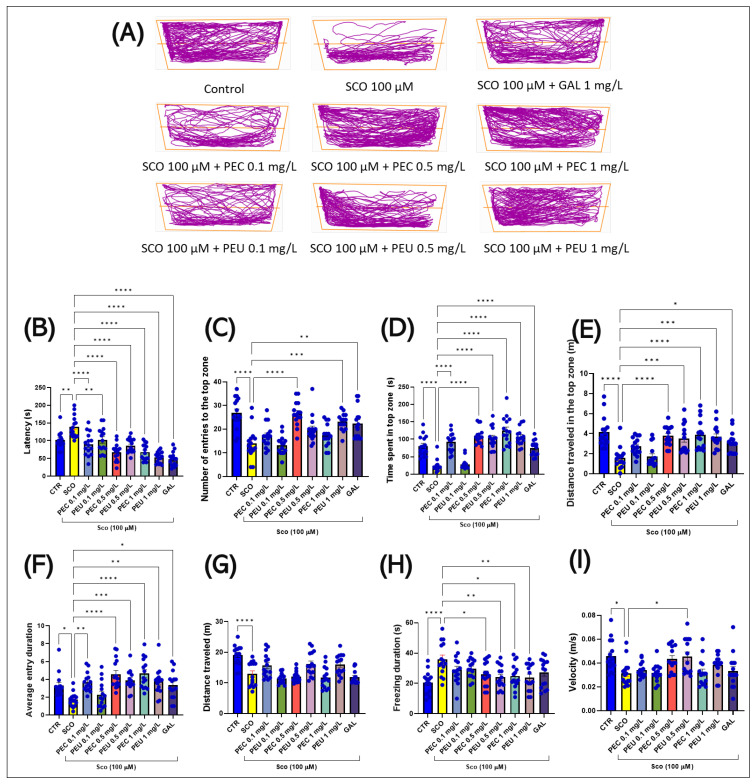
NTT results for PEC (0.1, 0.5, and 1 mg/L) and PEU (0.1, 0.5, and 1 mg/L). (**A**) A visual depiction of the swimming pattern in the NTT test; (**B**) Latency (s); (**C**) Number of entries to the top zone; (**D**) Time spent in top zone (s); (**E**) Distance traveled in the top zone (m); (**F**) Average entry duration (s); (**G**) Total distance traveled (m); (**H**) Freezing duration (s); and (**I**) Velocity (m/s). Data are presented as means ± SEM (n = 15 animals per group). * *p* < 0.05, ** *p* < 0.01, *** *p* < 0.001, and **** *p* < 0.0001 (Tukey’s post hoc analyses).

Figure 8A illustrates the differences in locomotor behavior in the NTT between the top and bottom zones of the tank. Groups treated with SCO (100 µM) preferred the bottom zone, suggesting an increased level of anxiety.

In the NTT, the one-way ANOVA indicated a significant impact of the treatment on various behavioral measures. Specifically, there was a notable effect on latency (s) [F (8, 126) = 18.67], (*p* < 0.0001) (Figure 8B), on the number of entries to the top zone [F (8, 126) = 12.73], (*p* < 0.0001) (Figure 8C), the time spent in the top of the tank [F (8, 126) = 26.96], (*p* < 0.0001) (Figure 8D), on the distance traveled in the top zone (m) [F (8, 126) = 9.175], (*p* < 0.0001) (Figure 8E), on the average entry duration (s) [F (8, 126) = 7.408], (*p* < 0.0001) (Figure 8F), on the total distance traveled (m) [F (8, 126) = 10.89], (*p* < 0.0001) (Figure 8G), on the freezing duration (s) [F (8, 126) = 4.073], (*p* < 0.0002) (Figure 8H), and on the velocity (s) [F (8, 126) = 4.540], (*p* < 0.0001) (Figure 8I).

Post hoc analyses using Tukey’s test indicated that acute treatment with SCO (100 µM) generated a pronounced anxiogenic response in zebrafish, as can be seen in Figure 8. The angiogenic response of the fish was evident in the increased latency (*p* < 0.01) for vertical exploration (Figure 8B), the decrease in the number of entries (*p* < 0.0001) in the top zone of the test tank (Figure 8C), the reduction significant difference in swimming time in the top zone (*p* < 0.0001) (Figure 8D), and by decreasing the distance traveled by fish (*p* < 0.0001) in the top zone (Figure 8E) compared to the control group. Simultaneous treatment with SCO (100 µM) also demonstrated a significant hypolocomotor effect on zebrafish, reflected by a reduction in average entry duration (*p* < 0.05, Figure 8F) and a marked decrease (*p* < 0.0001) in the total distance traveled (m) (Figure 8G). In addition, a considerable increase (*p* < 0.0001) in freezing duration (Figure 8H) and a significant reduction (*p* < 0.05) in average swimming speed (Figure 8I) were observed.

The anxiogenic effects induced by SCO (100 µM) for 30 min were ameliorated by the acute administration of PEC (0.1, 0.5, and 1 mg/L) and PEU (0.1, 0.5, and 1 mg/L) for 60 min, depending on the dose to which the fish were subjected. Both treatments caused an anxiolytic response in a concentration-dependent manner compared to the SCO treatment, managing to decrease the latency (s) [*p* < 0.0001 for PEC (0.1, 0.5, and 1 mg/L)] and [*p* < 0.01 for PEU (0.1 mg/L) and *p* < 0.0001 for PEU (0.5 and 1 mg/L)] (Figure 8B) and GAL 1 mg/mL (*p* < 0.0001), to increase the number of entries to the top zone [*p* < 0.0001 for PEC (0.5 mg/L)] and [*p* < 0.001 for PEU (1 mg/L)] (Figure 8C) and GAL 1 mg/mL (*p* < 0.01), to intensify time spent in the top zone [*p* < 0.0001 for PEC (0.1, 0.5, and 1 mg/L)] and [*p* < 0.0001 for PEU (0.1, 0.5, and 1 mg/L)] and GAL 1 mg/mL (*p* < 0.0001) (Figure 8D), and increase the distance traveled in the top zone [*p* < 0.0001 for PEC (0.5 mg/L) and *p* < 0.001 for PEC (1 mg/L)] and GAL 1 mg/mL (*p* < 0.05) (Figure 8E). In addition, the acute administration of PEC (0.1, 0.5, and 1 mg/L) and PEU (0.1, 0.5, and 1 mg/L) for one hour to zebrafish with SCO was able to efficiently restore their locomotor activity in NTT by amplifying average entry duration [*p* < 0.01 for PEC (0.1 mg/L) and *p* < 0.0001 for PEC (0.5 and 1 mg/L)] and [*p* < 0.01 for PEU (1 mg/L) and *p* < 0.001 for PEU (0.5 mg/L)] and GAL 1 mg/mL (*p* < 0.05) (Figure 8F) and the restriction of total freezing duration [*p* < 0.05 for PEC (0.5 and 1 mg/L)] and [*p* < 0.01 for PEU (0.5 and 1 mg/L)] (Figure 8G). Regarding the effects of the two natural extracts of *P. americana* fruits on the total distance traveled, no significant differences were observed between the group of fish subjected to acute treatment with SCO (100 µM) and the groups that were subsequently subjected to acute treatment with PEC and PEU. However, the post-hoc analyses developed by applying Tukey’s test indicated that there are obvious effects between the same concentrations of the extracts as follows: [*p* < 0.05 for PEC (0.1 mg/L) vs. PEU (0.1 mg/L), *p* < 0.05 for PEC (0.5 mg/L) vs. PEU (0.5 mg/L) and *p* < 0.05 for PEC (1 mg/L) vs. PEU (0.1 mg/L)] (Figure 8H). This may be due to the difference in the concentrations of bioactive compounds in the two extracts, due to the different methods of fruit processing before extraction. Moreover, post hoc analyses showed that PEC and PEU extracts are not able to change the mean speed of zebrafish pretreated with SCO in the NTT exploration tasks, except for PEU, in the concentration of (0.5 mg/L, *p* < 0.05) (Figure 8I).

### 3.6. Effects of PEC and PEU on the Zebrafish Anxiety in the Light/Dark Test

**Figure 9:**
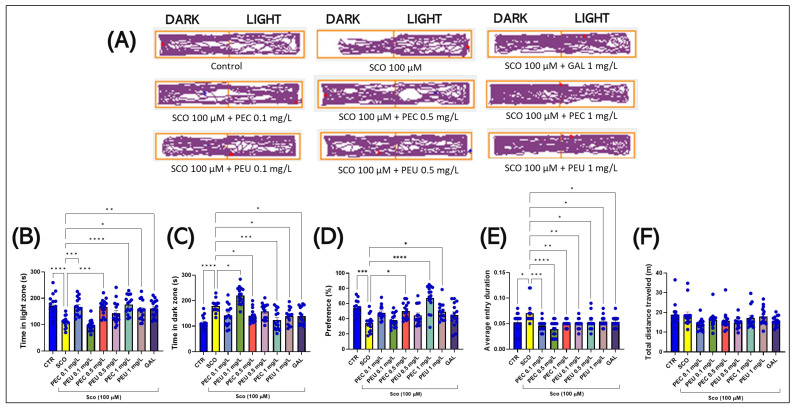
LDT results for PEC (0.1, 0.5, and 1 mg/L) and PEU (0.1, 0.5, and 1 mg/L). (**A**) A visual depiction of the swimming pattern in the LD test; (**B**) Time spent in the light zone (s); (**C**) Time spent in the dark zone (s); (**D**) Preference (%) (s); (**E**) Average entry duration (s) and (**F**) Total distance traveled (m). Data are presented as means ± SEM (n = 15 animals per group). * *p* < 0.05, ** *p* < 0.01, *** *p* < 0.001, and **** *p* < 0.0001 (Tukey’s post hoc analyses).

Figure 9 A illustrates the differences in locomotor behavior in the LDT between the lighted and dark zones of the tank. Groups treated with SCO (100 µM) preferred the dark zone, suggesting an increased level of anxiety.

In the LDT, the one-way ANOVA indicated a significant impact of the treatment on various behavioral measures. Specifically, there was a noticeable effect on the time spent in the light zone (s) [F (8, 126) = 10.37], (*p* < 0.0001) (Figure 9B), the time spent in the dark zone (s) [F (8, 126) = 14.80], (*p* < 0.0001) (Figure 9C), on the preference (%) [F (8, 126) = 9.424], (*p* < 0.0001) (Figure 9D), on the average entry duration (s) [F (8, 126) = 5.466], (*p* < 0.0001) (Figure 9E), and on the total distance traveled (m) [F (8, 126) = 2.240], (*p* = 0.0286) (Figure 9F).

Figure 9 shows that the zebrafish from the group treated exclusively with SCO (100 µM) showed a decrease in the time spent in the lighted zone (Figure 9B) at the expense of the time spent in the dark zone (s) (Figure 9C) compared with untreated fish (*p* < 0.0001). Simultaneously, the post hoc analysis data show that SCO negatively influences the preference (%) of fish for the light zone (*p* < 0.001, Figure 9D), thus suggesting the anxiogenic potential of SCO on zebrafish in LDT. Although SCO decreased the average entry duration (*p* < 0.05, Figure 9E), it did not influence the total distance traveled by the fish during the behavioral analysis session in the LDT.

Compared to the group of fish treated with SCO, the groups of fish treated with the two extracts showed an amplification of the time spent in the light zone (s) [*p* < 0.001 for PEC (0.1 and 0.5 mg/L) and *p* < 0.0001 for PEC (1 mg/L)] and [*p* < 0.05 for PEU (1 mg/L)] and GAL 1 mg/mL (*p* < 0.01) (Figure 9B), decreasing the time spent in the dark zone (s) [*p* < 0.05 for PEC (0.1 and 0.5 mg/L) and *p* < 0.001 for PEC (1 mg/L)] and [*p* < 0.05 for PEU (1 mg/L)] and GAL 1 mg /mL (*p* < 0.05) (Figure 9C), as well as the increase in preference (%) for the light zone [*p* < 0.05 for PEC (0.1 mg/L), *p* < 0.01 for PEC (0.5 mg/L) and *p* < 0.0001 for PEC (1 mg/L)] and [*p* < 0.05 for PEU (1 mg/L)] (Figure 9D) and also a decrease in average entry duration [*p* < 0.01 for PEC (1 mg/L), *p* < 0.001 for PEC (0. mg/L) and *p* < 0.0001 for PEC (0.5 mg/L)] and [*p* < 0.05 for PEU (0.5 and 1 mg/L) and *p* < 0.01 for PEC (0.5 mg/L)] and GAL 1 mg/mL (*p* < 0.05) (Figure 9E). Regarding total distance traveled (m), post hoc analyses found no difference between fish treated with SCO and those in the *P. americana* fruit extract groups (Figure 9F).

### 3.7. Effects of PEC and PEU on the Zebrafish Anxiety in the Novel Approach Test

**Figure 10:**
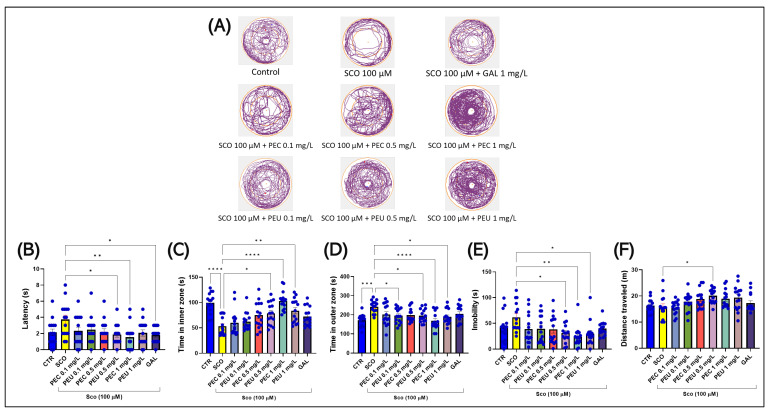
NAT results for PEC (0.1, 0.5, and 1 mg/L) and PEU (0.1, 0.5, and 1 mg/L). (**A**) A visual depiction of the swimming pattern in the NAT test; (**B**) Latency(s); (**C**) Time spent in the inner zone (s); (**D**) Time spent in the outer zone (s); (**E**) Immobility (s); and (**F**) Total distance traveled (m). Data are presented as means ± SEM (n = 15 animals per group). * *p* < 0.05, ** *p* < 0.01, *** *p* < 0.001, and **** *p* < 0.0001 (Tukey’s post hoc analyses).

Figure 10A illustrates the differences in locomotor behavior in the NAT between inner and outer zones. Groups treated with SCO (100 µM) preferred the outer zone, suggesting an increased level of anxiety.

In the NAT, the one-way ANOVA indicated a significant impact of the treatment on various behavioral measures. Specifically, there was a notable effect on the latency (s) [F (8, 126) = 2.436], (*p* = 0.0175) (Figure 10B), the time spent in the inner zone (s) [F (8, 126) = 9.545], (*p* < 0.0001) (Figure 10C), the time spent in the outer zone (s) [F (8, 126) = 4.277], (*p* < 0.0001) (Figure 10D), on the immobility periods (s) [F (8, 126) = 2.809], (*p* = 0.0067) (Figure 10D), and on the total distance traveled (m) [F (8, 126) = 3.249], (*p* = 0.0021) (Figure 10E).

Post-hoc analyses using Tukey’s test showed that acute treatment with SCO (100 µM) caused a significant anxiogenic response in zebrafish by reducing the time to explore the inner zone (s) near the figurine that was placed in the center of the tank test (*p* < 0.0001) and the significant increase in the exploration time of the dark zone (s) (*p* < 0.001), as illustrated in Figure 10C,D. Interestingly, however, there were no significant differences between the untreated fish in the group of control and fish subjected to the acute action of SCO in terms of latency (s) (Figure 10B), immobility time (s) (Figure 10E), and total distance traveled (Figure 10F) in NAT.

Regarding the groups of zebrafish that received acute pretreatment for 30 min with SCO and then 30 min with one of two fruit extracts of *P. americana*, the post hoc analyses highlighted the beneficial effects of the extracts on the latency period (s) of fish in NAT [*p* < 0.01 for PEC (1 mg/L)] and [*p* < 0.05 for PEU (0.5 mg/L)] and GAL 1 mg/mL (*p* < 0.05) (Figure 10B). Simultaneously, there was a significant effect of the two natural extracts compared to the group treated with SCO and on the total time spent by fish in the inner zone [*p* < 0.0001 for PEC (1 mg/L)] and [*p* < 0.05 for PEU (0.5 mg/L) and *p* < 0.01 for PEU (1 mg/L)] and outer zone [*p* < 0.001 for PEC (1 mg/L)] and [*p* < 0.05 for PEU (0.5 and 1 mg/L)] (Figure 10C). The post-hoc analysis also revealed a significant positive impact of the two extracts on the immobility time of the fish in NAT, compared to the fish treated only with SCO; thus, the treatment with PEC managed to decrease the immobility time in the concentration of 1 mg/ L (*p* < 0.01, Figure 10E), while PEU treatment was able to reduce immobility time in concentrations of 0.5 and 1 mg/L (*p* < 0.05, Figure 10E). However, post hoc analysis showed only a statistically significant difference between fish treated with the two extracts and those in the SCO group. Thus, only PEU at a concentration of 0.5 mg/L presented a significant effect on total distance traveled (m) in NAT (*p* < 0.05, Figure 10F).

### 3.8. Correlation of Time Spent by Fish in the Upper, Illuminated, and Inner Zone

**Figure 11:**
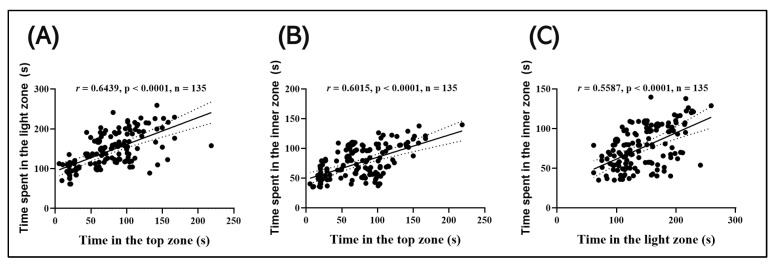
Correlation of time spent by zebrafish in the top (NTT), light (LDT), and inner (NAT) zones; (**A**) Time spent in the top zone (s) vs. time spent in the light zone (s), (**B**) Time spent in the top zone (s) vs. time spent in the inner zone (s), (**C**) Time spent in the light zone (s) vs. time spent in the inner zone (s) (n = 135 fish), (Pearson correlations).

Figure 11 shows the intercorrelations between the behavioral measures of both control and SCO-treated fish, as well as fish pretreated with SCO and then acutely treated with *P. americana* fruit extracts. A strong positive correlation was observed between the time spent by zebrafish on the top zone (NTT) and the time spent by zebrafish in the light zone (LDT) (*r* = 0.6439; *p* < 0.0001; n = 135) (Figure 11A), as well as between the time spent by zebrafish in the top zone (NTT) and the time spent by zebrafish in the inner zone (NAT) (*r* = 0.6015; *p* < 0.0001; n = 135) (Figure 11B). There was also a significant positive correlation between the time spent by zebrafish in the light zone (LDT) and the time spent by zebrafish in the inner zone (NAT) (*r* = 0.5587; *p* < 0.0001; n = 135) (Figure 11C).

**Figure 12:**
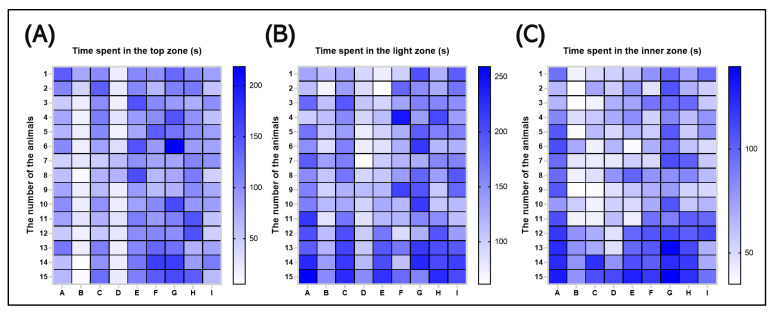
Representative heatmaps illustrating (**A**) Time spent in the top zone (s), (**B**) Time spent in the light zone (s), (**C**) Time spent in the inner zone (s) (n = 15 fish). At the bottom: A—Control; B—SCO (100 µM); C—PEC 0.1 mg/L; D—PEU 0.5 mg/L; E—PEC 0.5 mg/L; F—PEU 0.1 mg/L; G—PEC 1 mg/L; H—PEU 1 mg/L; I—GAL 1 mg/mL.

Individual fish values for time spent in the top zone (NTT), time spent in the light zone (LDT), and time spent in the inner zone (NAT) for zebrafish analyzed from the control and SCO groups, as well as acutely pretreated with SCO and then subjected to acute treatment with *P. americana* fruit extracts are shown in Figure 12. These values highlight the behavioral differences between the 15 analyzed batches.

The anxiolytic effects of PEC and PEU may be mainly due to the synergistic effects of their main compounds, such as epicatechin, naringenin, and isoquercetin (for PEC), as well as naringenin, ferulic acid, and protocatechuic acid (for PEU). A growing number of studies suggest that these compounds have the potential to reduce anxiety through various mechanisms, including modulating neurotransmitters and reducing inflammation and oxidative stress.

### 3.9. Effects of PEC and PEU on Zebrafish Spatial Memory in the Y-Maze

**Figure 13:**
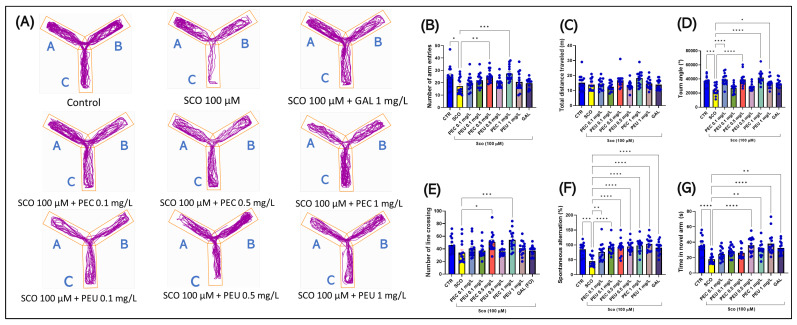
Y-maze results for PEC (0.1, 0.5, and 1 mg/L) and PEU (0.1, 0.5, and 1 mg/L). (**A**) A visual depiction of the swimming pattern in the Y-maze test; (**B**) Number of arm entries; (**C**) Total distance traveled (m); (**D**) Turn angle (°); (**E**) Number of line crossings; (**F**) Spontaneous alternation (%); (**G**) Time spent in the novel arm (s). A—Starting arm, B—Other arm, C—New arm. Data are presented as means ± SEM (n = 15 animals per group). * *p* < 0.05, ** *p* < 0.01, *** *p* < 0.001, and **** *p* < 0.0001 (Tukey’s post hoc analyses).

In the Y-maze, the one-way ANOVA indicated a significant impact of the treatment on various behavioral measures. Specifically, there was a notable effect on the number of arm entries [F (8, 126) = 4.591], (*p* < 0.0001) (Figure 13B), on the total distance traveled (m) [F (8, 126) = 3.279], (*p* = 0.0020) (Figure 13C), on the turn angle (°) [F (8, 126) = 10.44], (*p* < 0.0001) (Figure 13D), on the number of line crossing [F (8, 126) = 4.611], (*p* < 0.0001) (Figure 13E), spontaneous alternation (%) [F (8, 126) = 8.796], (*p* < 0.0001) (Figure 13F), and on the time spent in the novel arm (s) [F (8, 126) = 7.140], (*p* < 0.0001) (Figure 13G).

Post hoc analyses using the Tukey test indicated that keeping zebrafish in a solution of SCO (100 µM) for half an hour before performing the test session in the Y-maze paradigm negatively affected curiosity and desire to fully exploit the test tank, as can be seen in Figure 13B,D (*p* < 0.001); however, our analysis found no difference between fish in the control group and those in the SCO group regarding total distance traveled (Figure 13C). In addition, SCO negatively affected the spatial memory and short-term memory of fish by decreasing spontaneous alternation (*p* < 0.001, Figure 13F) and time spent in the novel arm (s) (*p* < 0.0001, Figure 13G).

Regarding the effects of PEC and PEU on the locomotor activity of amnesic zebrafish, Tukey’s post hoc analyses revealed that only PEC treatment in the concentration of 0.5 mg/L (*p* < 0.01) and the concentration of 1 mg/L (*p* < 0.001), could restore the number of arm entries (Figure 13B). At the same time, none of the two applied treatments showed a statistically significant effect on the total distance traveled (Figure 13C). However, both treatments showed a significant positive effect on turn angle (Figure 13D). More precisely, PEC was able to restore the turn angle in all three analyzed concentrations [*p* < 0.0001, for PEC (0.1, 0.5 and 1 mg/L)]. In contrast, PEU proved to be less efficient, managing to restore turn angle only in the high concentration [*p* < 0.01, for PEU (1 mg/L)]. Therefore, PEC shows increased potential in restoring locomotor activity in amnesic zebrafish compared to PEU.

To assess cognitive processes such as learning and memory, including spatial memory in zebrafish, we used three essential measurements: number of line crossing, percentage of spontaneous alternation (%), and the time spent by fish in the novel arm of the Y-maze. The number of line crossings and the percentage of spontaneous alternation is a relevant index for the number of sequential entries in all three arms of the Y-maze. Alternation is considered successful when the animal visits all three arms consecutively, either clockwise or counterclockwise. According to previous research [59], animals show a natural tendency to favor a certain direction of rotation, and this preference can significantly influence the alternation behavior. Assessing the time spent by fish in the novel arm is critical to understanding temporary spatial memory and discrimination memory. Time spent in the novel arm can indicate the fish’s level of interest and exploratory ability, crucial aspects for assessing cognitive functions. Post hoc analyses show that of the two analyzed extracts, only PEC [*p* < 0.05 for PEC (0.5 mg/L) *p* < 0.01 for PEC (1 mg/L)] can restore the number of line crossings (Figure 13E). However, both extracts can intensify spontaneous alternation (%) [*p* < 0.01 for PEC (0.1 mg/L) and *p* < 0.0001 for PEC (0.5 and 1 mg/L)] and [*p* < 0.0001 for PEU (0.1, 0.5, and 1 mg/L)] and GAL 1 mg/mL (*p* < 0.0001) (Figure 13F). Moreover, PEC in the concentration of 1 mg/L (*p* < 0.01) and PEU (0.5, and 1 mg/L, *p* < 0.0001) and GAL 1 mg/mL (*p* < 0.01) significantly improved time in the novel arm (Figure 13G). Findings suggest that PEC improves locomotor activity and memory while attenuating SCO-induced cognitive impairment. In contrast, PEU has no impact on locomotion but outperforms PEC in improving memory in zebrafish.

### 3.10. Effects of PEC and PEU on the Zebrafish Recognition Memory in the Novel Object Recognition Test

**Figure 14:**
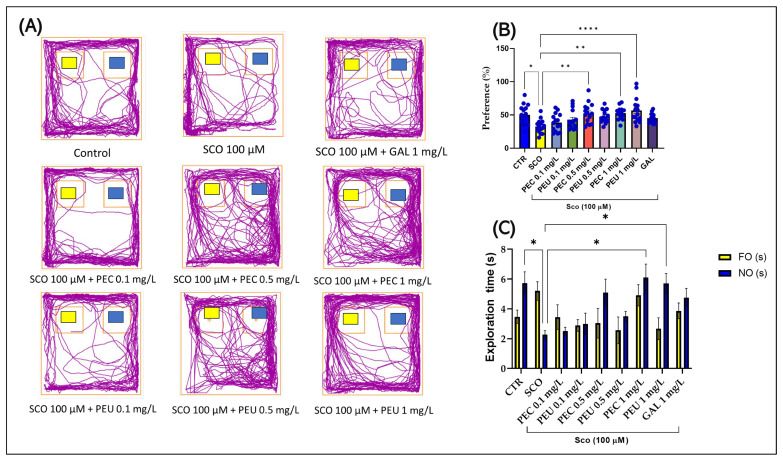
NOR results for PEC (0.1, 0.5, and 1 mg/L) and PEU (0.1, 0.5, and 1 mg/L). (**A**) A visual depiction of the swimming pattern in the NOR test; (**B**) Preference (%); (**C**) Exploration time (s); FO (yellow)—familiar object, NO (blue)—new object. (Data are presented as means ± SEM (n = 15 animals per group). * *p* < 0.05, ** *p* < 0.01 and **** *p* < 0.0001 (Tukey’s post hoc analyses).

In the NOR, the one-way ANOVA indicated a significant impact of the treatment on various behavioral measures. Specifically, there was a notable effect on the preference (%) [F (8, 126) = 4.974], (*p* < 0.0001) (Figure 14B) and on the exploration time (s) [F (8, 161) = 3.530], (*p* = 0.0009) (Figure 14C).

Post hoc analysis with Tukey’s test showed that animals treated with SCO had lower percentage preference and exploration time for NO compared to the control group (*p* < 0.05). Acute administration of PEC and PEU, especially at the dose of 1 mg/L, improved preference [*p* < 0.01 for PEC (0.5 and 1 mg/L) and *p* < 0.01 for PEU (1 mg/L)] (Figure 14B) and exploration time for NO [*p* < 0.05 for PEC and PEU (1 mg/L)] (Figure 14C), indicating improved memory.

### 3.11. Correlation of Time Spent by Fish in the Novel Arm of the Y-Maze and Their Preference for NO in Novel Object Recognition Test

**Figure 15:**
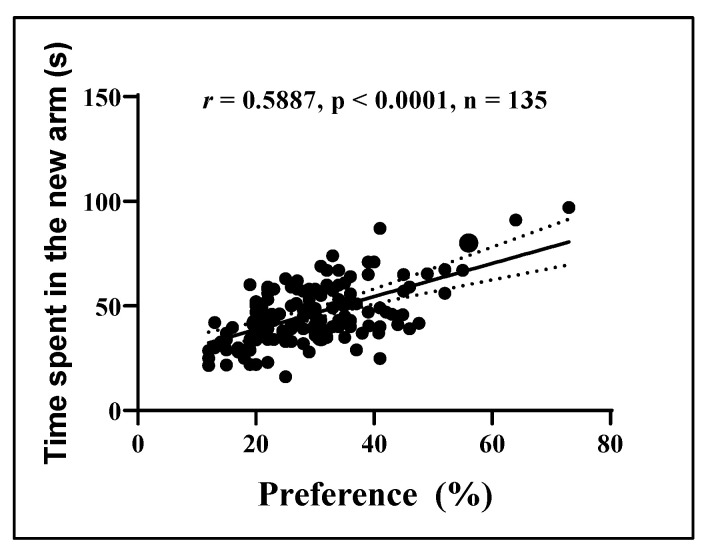
Correlation of time spent by zebrafish in the novel arm of the Y-maze and preference (%) in NOR (n = 120 fish), (Person correlations).

Figure 15 illustrates the associations between the behavioral endpoints of the control group of fish, the SCO-treated group, and the fish exposed to SCO followed by acute administration of *P. americana* fruit extracts. A clear positive relationship was identified between the time zebrafish spent in the novel arm of the Y-maze and their propensity to ON in the NOR test (*r* = 0.5887; *p* < 0.0001; n = 135).

**Figure 16:**
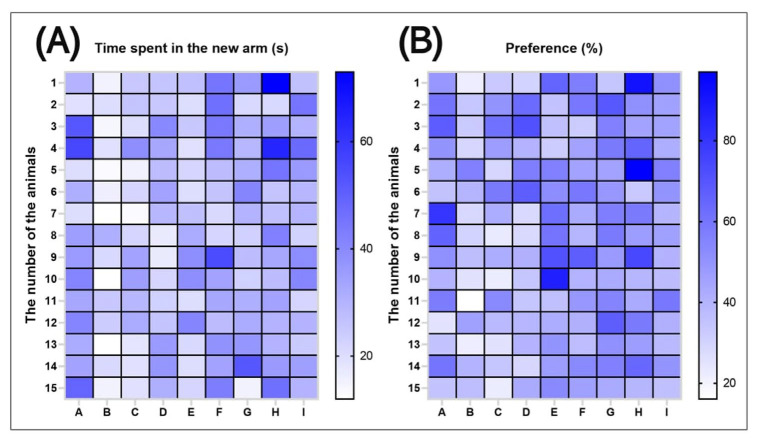
Representative heatmaps illustrating (**A**) Time spent in the novel arm (s) and (**B**) Preference (%) (n = 15 fish). At the bottom: A—Control; B—SCO (100 µM); C—PEC 0.1 mg/L; D—PEU 0.5 mg/L; E—PEC 0.5 mg/L; F—PEU 0.1 mg/L; G—PEC 1 mg/L; H—PEU 1 mg/L; I—GAL 1 mg/L.

Individual fish values for time spent in the novel arm of the Y-maze and their preference (%) in the NOR for zebrafish analyzed from the control and SCO groups, as well as fish pretreated acutely with SCO and then acutely treated with extracts of *P. americana* fruit are shown in Figure 16. These values highlight the behavioral differences between the 15 groups analyzed.

### 3.12. Effects of PEC and PEU on the AChE Activity

**Figure 17:**
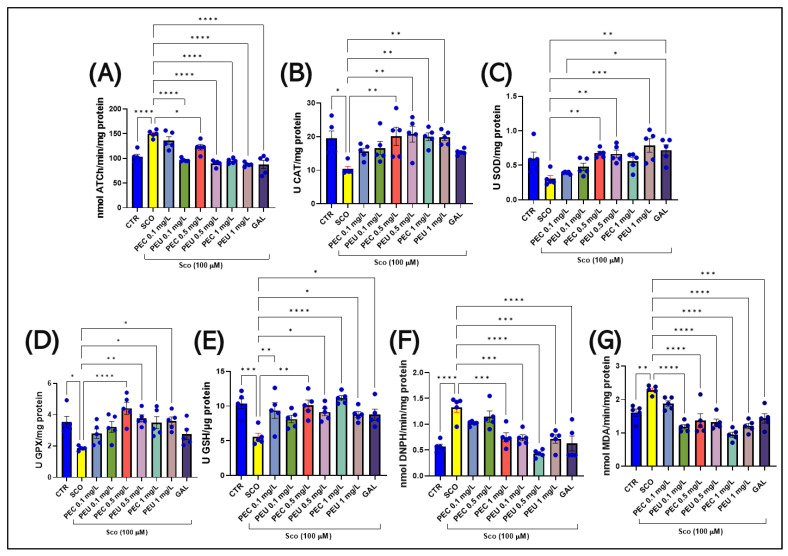
Effects of PEC (0.1, 0.5, and 1 mg/L) and PEU (0.1, 0.5, and 1 mg/L). (**A**) Acetylcholinesterase (AChE); (**B**) Catalase (CAT), (**C**) Superoxide dismutase (SOD); (**D**) Glutathione peroxidase (GPX) specific activities; (**E**) Total reduced glutathione content (GSH); (**F**) Protein carbonyl content; (**G**) Malondialdehyde (MDA) level. Values represent means ± SEM (n = 6) followed by Tukey’s post hoc analyses: * *p* < 0.05, ** *p* < 0.01, *** *p* < 0.001, and **** *p* < 0.0001.

Brain AChE activity was evaluated to understand how PEC and PEU may help SCO-induced memory deficits. One-way ANOVA showed that treatment had a significant effect on AChE activity (F (8, 36) = 21.92, *p* < 0.0001). Figure 17 shows that treatment of zebrafish with acute SCO at 100 μM resulted in a significant increase in AChE activity in the brain compared to the control group (*p* < 0.001). In contrast, zebrafish treated with SCO and PEC or PEU had reduced AChE activity [*p* < 0.01 for PEC (0.5 mg/L) and *p* < 0.0001 for PEC (1 mg/L)] and [*p* < 0.0001 for PEU (0.1, 0.5, and 1 mg/L)] and GAL 1 mg/mL (*p* < 0.0001) (Figure 17A), suggesting that both PEC and PEU exhibit anti-AChE effects.

### 3.13. Effects of PEC and PEU on the Brain Oxidative Stress

The impact of oxidative stress on cognition in AD is significant, being correlated with the degree of anxiety and amnesia; as a result, the impact of acute administration of PEC and PEU on oxidative stress in the zebrafish brain was evaluated. ANOVA showed significant treatment effects on CAT (F (8, 36) = 4.048, *p* = 0.0016), SOD (F (8, 36) = 6.176, *p* < 0.0001), GPX (F (8, 36) = 5.419, *p* = 0.0002), GSH (F (8, 36) = 5.456, *p* = 0.0002), carbonylated proteins (F (8, 36) = 12.13, *p* < 0.0001), and MDA (F (8, 36) = 13.69, *p* < 0.0001).

Tukey’s post hoc analyses showed a significant decrease in CAT activity (*p* < 0.05) caused by SCO compared to the control group (Figure 17B). Simultaneously, the acute treatment with SCO caused a decrease in SOD activity (*p* < 0.05) (Figure 17C), GPX (*p* < 0.05) (Figure 17D), and a decrease in reduced GSH content (*p* < 0.001) (Figure 17E). Moreover, SCO increased the amount of carbonylated protein (*p* < 0.0001) (Figure 17F) and enhanced the level of MDA (*p* < 0.001) (Figure 17G) in the brains of adult zebrafish compared to adult control fish.

Acute treatment with either of the two *P. americana* fruit extracts managed to restore the specific activity of CAT only at the higher concentrations (Figure 17B), [*p* < 0.05 for PEC (0.5 and mg/L) and [*p* < 0.05 for PEU (mg /L) and *p* < 0.01 for PEU (1 mg/L)] (Figure 17B). Regarding SOD activity, it could be modified positively in the middle concentration of PEC treatment (*p* < 0.01 for PEC (0.5 and 1 mg/L), as well as in the highest concentrations of PEC treatment PEU [*p* < 0.0001 for PEU (0.1, 0.5, and 1 mg/L)] (Figure 17A). Simultaneously, the extracts showed their antioxidant effect by improving GPX activity, a more pronounced effect being shown by PEC in the concentration of 0.5 mg/L (*p* < 0.0001) and PEU in the concentration of 0.5 mg/L (*p* < 0.01) but also in the maximum concentration [*p* < 0.05 for PEC and PEU (1 mg/L)] (Figure 17D). As for the reduced GSH content, post hoc analyses showed that there is a different pattern of action of the two extracts [*p* < 0.01 for PEC (0.1 mg/L), *p* < 0.001 for PEC (0.5 mg /L), and *p* < 0.0001 for PEC (1 mg/L)] and [*p* < 0.05 for PEU (0.1 and 0.5 mg/L)] (Figure 17E). Both PEC and PEU showed a similar effect on the amount of protein carbonyls, managing to significantly decrease their amount in the fish brain [*p* < 0.0001 for PEC (0.1, 0.5, and 1 mg/L) and *p* < 0.0001 for PEU (0.1, 0.5, and 1 mg /L)] (Figure 17F). Additionally, the two extracts proved to be efficient in regulating the level of MDA [*p* < 0.0001 for PEC (0.1, 0.5, and 1 mg/L) and *p* < 0.0001 for PEU (0.1, 0.5, and 1 mg/L)] (Figure 17G). Therefore, our data suggest that PEC and PEU can ameliorate the oxidative effects of SCO by improving the antioxidant defense of zebrafish by restoring the activities of SOD, CAT, and GPX, restoring the content of GSH and by decreasing the level of oxidative stress products (protein carbonyls and MDA).

## 4. Discussion

In the present investigation, the polyphenolic constituents of the two hydroethanolic extracts of *P. americana* fruit (PEU and PEC) were identified by the HPLC analytical technique. Our findings indicated that the predominant components of PEC included epicatechin, naringenin, isoquercetin, catechin, and protocatechuic acid.

The results reported by Marinas et al. [73] on *P. americana* fruit extracts obtained using 30 mL of 70% ethanol and 1.1310 g of fruit and an ultrasonic bath demonstrate the presence of epicatechin (0.39 µg/mL) and catechin (0.83 µg/mL). However, the authors detected quercetin instead of isoquercetin, likely due to the different extraction methods used. Marinas et al. [73] also reported a ferulic acid concentration of 0.12 µg/mL in *P. americana* berry extract and 0.98 µg/mL in leaves. Similarly, Bylka and Matławska [74] detected ferulic acid in *P. americana* leaves. Based on these studies, our *P. americana* berry extract shows a comparable chemical composition, which suggests similar antioxidant activity as reported by other authors.

In the raw fruit, a significant part of naringenin may be present as glycosides (naringin) or bound to other molecules such as cellulose or proteins. During the drying process, due to the structural and chemical changes that occur in the fruit, the glycosides can hydrolyze and release naringenin in its free form. This would explain why more naringenin is detected in the dried fruit. Naringenin may be more stable under drying conditions because the process reduces oxidative and hydrolytic activity that could affect flavonoids in the fresh fruit. In raw fruit, exposure to oxygen and moisture can lead to the degradation of naringenin or its conversion. Dried fruit, however, provides an environment that allows the preservation of naringenin in more stable forms available for extraction. The drying process can change the cellular structure of the fruit, making the cell walls more permeable and allowing the solvent to penetrate more efficiently and better extract bioactive compounds. In raw fruit, the cell walls are more unbroken, which can make extraction less efficient [75].

The raw fruit contains a large amount of active enzymes that can degrade phenolic compounds, including ferulic acid. These enzymes (e.g., esterases, peroxidases, or phenoloxidases) may be more active in the fresh state of the fruit and may break down ferulic acid before extraction, thus preventing its detection by HPLC. On the other hand, in the case of dried fruit, the dehydration process reduces enzyme activity. Drying inactivates most enzymes, preventing the degradation of phenolic compounds and thus allowing ferulic acid to be extracted and detected. Additionally, the drying process can induce chemical changes that release ferulic acid from its bound forms. For example, ferulic acid may be bound to polysaccharides or other molecules in the raw fruit and released during drying [76].

Epicatechin is a compound that is relatively sensitive to heat, light, and oxygen. During the drying process, these conditions can lead to oxidation and degradation of epicatechin. Drying at high temperatures or prolonged exposure to air can induce oxidative changes in the structure of epicatechin, leading to the formation of dimers (such as procyanidins), which are not detectable as epicatechin by HPLC. In contrast, in the raw fruit, epicatechin is stable and protected under a more water-laden environment. Epicatechin, like other flavonoids, can undergo polymerization during drying, forming larger and more complex compounds such as condensed tannins or procyanidins. These compounds are more difficult to detect as free epicatechin on HPLC. In the raw fruit, epicatechin is present in monomeric form, and polymerization does not occur to the same extent, making epicatechin detectable in HPLC analysis [77]. Thus, according to the findings presented in the existing literature, the chemical composition of the ethanol extracts of *P. americana* fruits aligns with the results previously reported in the literature.

Regarding the safety of *P. americana* extracts for pharmaceutical purposes, opinions are divided due to their potentially toxic effects. At a dose of 1 g, the dried rhizome of *P. americana* functions as an emetic and purgative agent. At low doses ranging from 60 to 100 mg daily, both the root and the fruit have been used in the management of rheumatism and in increasing the effectiveness of the immune system [78,79]. The foliar and underground components of *P. americana* have been used in traditional medicinal practices to relieve chronic rheumatism and arthritis, in addition to serving as an emetic and purgative [80]. Furthermore, the plant has been used to manage various conditions, including respiratory disorders, such as catarrh; gynecological disorders, such as dysmenorrhea; viral infections, such as mumps; and skin infections, such as edema, skin neoplasms, ringworm, and scabies, as well as other conditions, such as tonsillitis and syphilis [81]. The young leaves, called “salad poke”, are used in food, preserved, and sold commercially. In addition, the juice extracted from the berries has been used as an ink, dye, and pigmenting agent in winemaking [29]. While *P. americana* can be safely ingested when meticulously prepared using boiling techniques, consumption of unprocessed plant material or undercooked culinary preparations can lead to considerable toxicosis [82]. The botanical specimen is characterized by the presence of phytolaccin, a strong irritant for the gastrointestinal system that, in humans, can cause a series of symptoms, from a burning sensation in the digestive tract to the occurrence of severe hemorrhagic gastritis [83]. The plant also contains various harmful compounds, including pokeweed mitogen, which is a unique polypeptide that possesses the ability to inhibit ribosomal RNA synthesis, in addition to a series of saponins that induce gastrointestinal distress characterized by nausea, emesis, and diarrhea, frequently accompanied by profuse foaming [29]. Concentration levels of these compounds fluctuate throughout the plant’s growing season, being more abundant in the leaves and less abundant in the fruits [84]. Despite these symptoms, ingestion of *P. americana* is considered non-lethal, usually resolving within a few days with appropriate care measures, with ingestion of up to 10 fruits at one meal being considered harmless for adults [28].

The compounds identified in the two *P. americana* extracts were finally analyzed in comparison with SCO and GAL by in silico methods of predicting pharmacokinetic properties, such as absorption, distribution, metabolism, excretion, and toxicity, using the SwissAdme [34], Molinspiration [39], ProToX-III [44], AdmetLab 3.0 [43], PKCSM [42], and PASS [45] online platforms. The results of the ADMET profile show that these compounds exhibit different pharmacokinetic properties, with generally high intestinal absorption, low skin permeability, and limited ability to cross the blood/brain barrier (except for GAL and ferulic acid). Only SCO and GAL are metabolized by CYP3A4, while naringenin and myricetin act as inhibitors of CYP1A2, suggesting potential drug–drug interactions. The excretion profiles indicate moderate elimination, with only GAL identified as a renal OCT2 substrate, which may influence renal clearance. Toxicity assessments show relatively low risks of cardiac arrhythmias (myricetin), but certain compounds (e.g., isoquercetin, GAL) present higher risks of hepatotoxicity, nephrotoxicity, and genotoxicity, limiting their long-term safety for use. Although immunotoxicity is low, high AMES and genotoxicity scores for some compounds (e.g., isoquercetin) suggest potential risks of DNA damage, requiring careful monitoring of doses and safety in therapeutic applications. Additionally, most compounds identified in the two extracts present lower toxic potential compared to GAL. Simultaneously, all compounds identified in PEC and PEU show antioxidant, anti-inflammatory, and potential anti-dementia effects (e.g., chlorogenic acid). In contrast, SCO shows the potential to induce dementia.

The effects of PEC and PEU on memory and anxiety were examined using a zebrafish model (Tubingen strain) with cognitive impairment induced by SCO (100 μM). Three doses of PEC and PEU (0.1, 0.5, and 1 mg/L) were chronically administered to zebrafish treated with SCO, which led to behavioral changes observed in in vivo tests. During the treatment with PEC and PEU, no fish exposed to the treatment died or showed signs of toxicity. This may be due to the fact that following ethanol processing, certain toxic substances present in *P. americana* fruits are neutralized or to the different mode of exposure of the treatment (by immersion) compared to gastrointestinal administration. This may also be because the concentrations used in this study were very low (<1 mg/L). Results indicated that both PEC and PEU treatment reduced SCO-induced anxiety in behavioral tasks (NTT, NAT, and LDT). These results are in agreement with those in the literature, where SCO is described as an anticholinergic drug commonly used to treat motion sickness and as an antispasmodic agent [85]. It works by inhibiting mAChRs, thus affecting the CNS and peripherals. Studies have shown that SCO can induce cognitive impairment, anxiety symptoms, and electrophysiological changes, which resemble those observed in AD, especially in experimental animal models [86,87]. Additionally, in humans, acute administration of SCO can lead to confusion, anxiety, agitation, and irritability [88]. In zebrafish, SCO showed an anxiogenic effect, confirming its utility in anxiety research [89]. Furthermore, multiple studies have associated the preference of zebrafish for the dark zone following treatments with anxiogenic substances [48,75,76]. Interestingly, however, SCO hydrobromide in high concentrations (800 µM) induces anxiolytic effects in zebrafish [56]. This may be because, unlike SCO, SCO hydrobromide does not cross the BBB due to its quaternary ammonium salt moiety and thus does not induce direct adverse effects on the CNS [90].

There are no scientific reports regarding the effects of *P. americana* on anxiety, but the biocompounds present in PEC and PEU are well documented. Stringer et al. [91] demonstrated that daily administration of (-) epicatechin (4 mg/day) to adult male C57BL/6 mice resulted in a significant reduction in anxiety as measured by standard behavioral tests such as the elevated plus maze (EPM) and open field test (OFT). The authors also found an increase in cortical tyrosine hydroxylase expression and downregulation of monoamine oxidase-A, indicating a change in monoaminergic pathways that could contribute to anxiety reduction. In addition, administration of (-) epicatechin induced increased levels of BDNF, pro-BDNF, and pAkt activity in the hippocampus of animals. Therefore, the anxiolytic effects of (-) epicatechin are probably mediated by a synergy between the modulation of neurotrophic and monoaminergic pathways without necessarily involving hippocampal neurogenesis. Kang et al. [92] demonstrated that (−)-epicatechin induces significant changes in the expression of 1001 genes, 241 miRNAs, and 167 long non-coding RNAs, which regulate processes related to neurofunction, inflammation, and cell signaling, thus contributing to the reduction of anxiety-induced behaviors induced by obesity.

Abdelkawy et al. [93] showed that naringenin (50 mg/kg), administered either alone or in combination with liraglutide, attenuates depressive-like behavior in mice by improving neurogenesis and reducing neuroinflammation, which contributes to the restoration of monoamine levels comparable to those obtained with escitalopram. Oladapo et al. [94] indicate that naringin, a flavonoid isolated from citrus, attenuates social stress-induced behaviors by increasing GAD67 levels and inhibiting AChE, thereby reducing oxidative, nitrergic stress and neuroinflammation in affected brain regions and by modulating the GABAergic system, which is involved in mood regulation and anxiety. Quercetin, administered at low to moderate doses, demonstrated positive effects on shoaling behaviors and anxiety, helping to reduce oxidative stress, neuronal apoptosis, and neuroinflammation in a zebrafish model [95]. Isoquercetin, a glycosidic derivative of quercetin, exhibits increased bioavailability and is more rapidly absorbed, having the ability to triple the plasma levels of quercetin metabolites in rats [96]. The antidepressant effects of isoquercetin are due to the reduction of oxidative stress and by blocking 5-HT1A receptors, as well as by inhibiting the Nrf2 factor and by stopping the NOX4/ROS/NF-κB pathway [97].

Deng et al. [98] showed that ferulic acid can effectively reduce symptoms of anxiety and depression in mice by positively influencing the gut microbiome and microbial metabolism. Dong et al. [99] reveal the antidepressant effects of ferulic acid through various mechanisms, including neurotransmitter regulation, inhibition of neuroinflammation, and promotion of neurogenesis. Sur et al. [100] demonstrated that protocatechuic acid administered (100 or 200 mg/kg) once daily for 14 days significantly reduced fear-related behaviors and regulated the imbalance of serotonin and norepinephrine. Thakare et al. [101] showed that protocatechuic acid exhibits an important antidepressant mechanism by ameliorating oxidative stress and stimulating the endogenous antioxidant system, as well as by improving monoamine levels in the brain.

Simultaneously, PEC and PEU, both in concentrations of 0.1, 0.5, and 1 mg/L, improved memory deficits induced by acute treatment with SCO in pregnancies (Y-maze and NOR). Our findings line up exactly with results obtained by other research teams, illustrating the impact of SCO on learning and memory processes in animals. Administration of SCO to rats by intraperitoneal injection was linked to decreased performance in passive avoidance tasks and elicited neurotoxic effects [102]. Richetti et al. [103] demonstrated that SCO caused significant memory deficits in zebrafish in the inhibitory avoidance behavioral task. These results were subsequently confirmed in various behavioral paradigms [60,104]. Simultaneously, several reports have found that SCO negatively affects spatial memory and short-term memory in zebrafish [104,105].

Obulesu et al. [106] showed that naringin improves behavior and cognitive functions in animal models of epilepsy induced by kainic acid as well as in Huntington’s disease induced by 3-nitropropionic acid, suggesting the potential of naringin to modulate neurodegenerative processes and support cognitive health. Chiu et al. [107] investigated the ex vivo neuroprotective effects of naringin through long-term potentiation (LTP) on organotypic hippocampal slice cultures, as well as its in vivo impact (100 mg/kg/day for 20 days) on injection-impaired cognitive functions bilateral with Aβ peptides in the CA1 subregion of the hippocampus. The results of the study showed that naringin increased the excitatory field postsynaptic potential (fEPSP) in hippocampal slices and attenuated Aβ-induced blockade, thereby improving object recognition memory, passive avoidance memory, and spatial recognition memory in Aβ-injected rats. At the molecular level, naringin reduced cyclooxygenase-2, inhibited Bax activation, and upregulated the expression of neurotrophic proteins, suggesting promising therapeutic potential in reducing Aβ-induced neuronal inflammation and apoptosis through BDNF/TrkB/CREB signaling.

Yang et al. [108] elucidated that isoquercetin exhibits considerable neuroprotective efficacy in both in vitro and in vivo paradigms of AD. In PC12 cell models subjected to LPS, isoquercetin attenuated the generation of reactive oxygen species (ROS) and proinflammatory cytokines, simultaneously improving antioxidant values. In an in vivo study using Wistar rats, isoquercetin facilitated improvements in cognitive performance and decreased Aβ peptide and carbonyl protein concentrations while increasing BDNF synthesis and AChE enzyme activity. Rossato et al. [109] reported that nano-encapsulated ferulic acid (FA-Nc) improved memory and reduced anxiety in rats treated with d-galactose, which mimics the aging process. FA-Nc maintained the levels of essential neurotransmitters (ACh, glutamate, and GABA) in the brain. Kim et al. [110] investigated the neuroprotective effects of protocatechuic acid using a mouse model of SCO-induced memory impairment. Administration of protocatechuic acid at a dose of 10 mg/kg or higher for two weeks significantly improved learning and memory performance, reduced oxidative stress markers, increased expression of antioxidant proteins in the hippocampus, and attenuated histological brain damage. These findings suggest that protocatechuic acid could serve as a promising dietary supplement for reducing cognitive deficits associated with neurodegenerative diseases by combating oxidative stress.

Brain AChE activity was evaluated to understand how PEC and PEU may help SCO-induced memory deficits. ACh is essential for learning and memory, and decreased levels are associated with various neurodegenerative conditions, including AD. AChE inhibitors, which prevent the breakdown of ACh, are used to improve symptoms and slow the progression of AD and Parkinson’s disease dementia [111,112]. Synthesis and aggregation of Aβ contribute to AD, and dual inhibitors for AChE and Aβ have been developed. Cholinergic neurons in the basal forebrain are crucial for cognitive function, and their degeneration in AD leads to decreased ACh levels and memory decline. Restoration of the cholinergic system is an important therapeutic strategy. AD causes irreversible damage and represents a significant burden, underscoring the urgent need for effective treatment [111,113]. Our results show that both PEC and PEU can modulate AChE activity, as it was disrupted following acute exposure to SCO. Previously, *P. americana* L. showed a notable inhibitory effect on AChE, evaluated by in vitro methodologies. The methanol extract derived from the foliage of the plant demonstrated considerable inhibitory potency against AChE, obtaining an activity measurement of 2.05 mg GAL equivalent/g of extract [114]. The authors suggest that it may serve as a valuable source of bioactive compounds with cholinesterase inhibition attributes, a facet of considerable relevance in the formulation of cosmetic or pharmaceutical products, and with implications for the management of neurodegenerative disorders related to cholinesterase activity [114].

In addition, we also examined the antioxidant potential of PEC and PEU in the zebrafish model induced by SCOP by evaluating the specific activities of defense antioxidant enzymes and the degree of oxidation of lipids and proteins. Several studies indicate the importance of ROS and oxidative stress in the development of AD due to their damaging impact on molecules, especially proteins. Recent examinations of post-mortem AD brain samples show oxidative damage to enzymes related to energy metabolism, neurotransmitter-related proteins, mitochondrial proteins, and proteasomal components [115]. Oxidative imbalance in amyloid plaques leads to increased lipid peroxidation, protein oxidation, and nucleic acid oxidation. Decreased antioxidants such as uric acid, albumin, and vitamins are also linked to AD. Oxidative stress can promote Aβ peptide production and tau protein phosphorylation, contributing to the pathogenesis of AD [116]. In AD patients, increased levels of ROS are accompanied by increased levels of reactive nitrogen species (RNS), such as peroxynitrite, contributing to Aβ accumulation and τ phosphorylation. Increased NOS expression is linked to Aβ deposits, indicating that Aβ aggregates can trigger NOS to generate nitric oxide, leading to 3-NT formation and the development of oxidative stress [117,118,119]. Our results indicate that PEC and PEU exhibit antioxidant properties and alleviate oxidative stress caused by acute exposure to SCO treatment. This is achieved by increasing the activities of SOD, CAT, and GPX enzymes, GSH content, and reducing levels of MDA and carbonylated proteins. Previously, numerous studies have highlighted the increase in oxidative stress following exposure to SCO, both in zebrafish [104] as well as in mice [120], as well as in rats [121]. Additionally, the biocompounds present in the investigated *P. americana* fruit extracts have previously been associated with strong antioxidant effects. Thus, epicatechin demonstrated protective effects by reducing oxidative stress and decreasing levels of ROS and lipid peroxidation while increasing GSH levels. Epicatechin also improved mitochondrial function and protected mitochondria against homocysteine-induced damage, suggesting a therapeutic potential in the prevention of neurodegenerative disorders [122]. Naringenin can also improve cognitive function, as demonstrated by behavioral tests, including the object recognition test and the Morris water maze test. Simultaneously, naringenin inhibits oxidative stress by reducing the generation of reactive oxygen species and MDA, increasing the activity of antioxidant enzymes (SOD and GPX) in the hippocampus. In addition, it reduces inflammation by decreasing pro-inflammatory cytokines (IL-1β, IL-6, and TNF-α) and increasing anti-inflammatory ones (IL-10 and IL-4) while promoting N-methyl-D-aspartate receptor signaling, which suggests therapeutic potential in the management of cognitive dysfunction [123]. Isoquercetin also demonstrated non-toxic effects on PC12 cells, significantly reducing the production of ROS and pro-inflammatory cytokines. In rat experiments, isoquercetin-treated groups showed significant improvements in cognitive function and had reduced levels of Aβ peptides and inflammatory mediators. Therefore, isoquercetin may prevent neurochemical and neurobehavioral changes associated with AD through antioxidant and anti-inflammatory mechanisms [108]. Similarly, ferulic acid improves the survival rate of palmitic acid-treated HT22 cells, inhibits cell apoptosis, and reduces oxidative stress through the IRS1/PI3K/AKT/GSK3β signaling pathway. Ferulic acid treatment also improved learning and memory in HFD-fed mice, suggesting that ferulic acid may be developed as a potential agent for the treatment of high-fat diet-induced cognitive impairment [124]. Additionally, protocatechuic acid reduces the content of Pb in the body of rats, including blood, bone, brain, liver, and kidney, and inhibits oxidative damage by increasing the activity of antioxidant enzymes, such as SOD and GPX. Protocatechuic acid also improves learning and memory by increasing neurotransmitter levels and maintaining the normal function of N-methyl-d-aspartate receptors (NMDARs), thereby activating relevant signaling pathways [125].

Wang et al. [126] evaluated the impact of myricetin on SH-SY5Y neuronal cells treated with oligomer Aβ_42_ and on 3xTg mice by behavioral assays, analyzing the levels of proteins involved in AD pathology. The results showed that myricetin improves memory and learning in mice, reduces phosphorylation of tau and pre- and postsynaptic proteins, and decreases oxidative stress and mitochondrial dysfunction through GSK3β and ERK 2 signaling pathways. Additionally, catechins can inhibit pro-oxidant enzymes such as NADPH -oxidase and can modulate the interaction of ligands with receptors, such as tumor necrosis factor-alpha (TNF-α), thus contributing to the overload of pathways associated with oxidative stress and inflammation. These compounds also influence the activities of redox-sensitive transcription factors such as NF-κB and AP-1, which play an essential role in the pathological response to oxidative stress [127]. Apart from that, Sapturi et al. [128] demonstrated that chlorogenic acid improves memory function in diabetic rats by up-regulating the mRNA expression of SOD1, SOD2, and Bcl-2 genes, indicating the neuroprotective potential of chlorogenic acid, helping to ameliorate the negative effects of hyperglycemia on memory.

The nutrients and bioactive constituents present in the ethanolic extracts of *P. americana* can thus be designated as “neurohormetic nutrients” due to their ability to influence brain health through dose-dependent mechanisms, as outlined by the principle of neurohormesis. [129,130]. This concept emphasizes that exposure to low or moderate levels of bioactive stimuli activates cellular adaptive pathways, thereby enhancing neuronal resilience and protecting against cognitive decline and oxidative stress. These bioactive compounds, such as flavonoids (e.g., epicatechin, catechin, naringenin) and phenolic acids (e.g., ferulic acid), support neuroprotection by modulating AChE activity and stimulating antioxidant pathways, including SOD, CAT, and GPX. Through these mechanisms, they effectively regulate oxidative stress, as evidenced by the reduction of oxidative markers such as carbonylated proteins and MDA, thereby contributing to improved cognitive functions (learning and memory) and emotional states. These compounds enhance brain function by activating neurotransmitters and synaptic receptors. These effects are dose-dependent, playing a role in preventing or mitigating impairments in plasticity and memory. In zebrafish (*Danio rerio*), these mechanisms may have direct applications in the study of neurodegenerative diseases. Animal models suggest that flavonoids and other plant-derived bioactive compounds improve neuronal homeostasis, protect the BBB, and reduce oxidative stress and neuroinflammation—factors involved in the pathogenesis of neurodegenerative diseases. Furthermore, gut/brain axis interactions mediated by functional nutrients such as polyphenols and probiotics can improve compound bioavailability and cognitive function, preventing systemic inflammation and neurological disorders [129]. On the other hand, high doses of nutrients or dietary supplements can become neurotoxic, inhibiting neuroprotective pathways and promoting the development of neurological disorders such as AD and anxiety [130].

The current limitations of the study include the variability of individual responses to different doses of functional extracts and the need for further research to better understand the precise mechanisms through which neurohormetic nutrients influence cognitive functions and protect against neurodegenerative diseases. Additionally, future studies should assess the long-term effects of these nutrients to evaluate their safety and efficacy in therapeutic applications. Future research directions could involve testing the effects of PEC and PEU at higher doses and over longer durations, as well as using additional animal models to validate these findings. Moreover, it would be relevant to explore the detailed molecular mechanisms by which these neurohormetic nutrients enhance brain function and synaptic plasticity in the context of cognitive and neurodegenerative disorders.

## 5. Conclusions

Two hydroethanolic extracts developed from raw (PEC) and dried (PEU) fruits of *P. americana* were investigated for their impact on anxiety-like behavior, memory, oxidative stress, and cholinergic status in zebrafish treated with scopolamine (SCO). The HPLC analysis of the natural extracts revealed epicatechin as the main phenolic compound in PEC and naringenin in PEU.

Our data suggest that both PEC and PEU exhibit anxiolytic effects, being able to attenuate the anxiogenic effects induced by SCO in behavioral tasks (NTT, LDT, and NAT) performed on zebrafish. In addition, both extracts improved the cognitive performance of amnesic zebrafish in behavioral tasks (Y-maze and NOR). At the same time, the two extracts demonstrated the ability to improve cholinergic deficits caused by SCO, having a strong inhibitory effect on AChE at all three tested concentrations (0.1, 0.5, and 1 mg/L). In addition, PEC and PEU strengthened the antioxidant system of fish by increasing the activity of antioxidant enzymes and reducing markers of lipid and protein peroxidation. Therefore, both PEC and PEU extract managed to regulate the activity of AChE and improve the activity of antioxidant enzymes in the brain of zebrafish treated with SCO and to decrease markers of protein oxidation (protein carbonyls) and lipid peroxidation (MDA). However, PEU proved to be more effective in ameliorating the anxiogenic effects induced by SCO, as can be observed in behavioral paradigms (LDT and NAT), while PEC proved to be more effective in restoring spatial memory (by increasing the time to explore the novel arm in Y-maze) and improving the recognition memory of zebrafish treated with SCO (by restoring preference (%) in NOR). Thus, the fruit extracts of *P. americana* could represent a viable therapeutic alternative for the amelioration of anxiety and cognitive deficits associated with amnesia.

## Figures and Tables

**Table 1:** SMILES data for SCO, GAL, catechin, epicatechin, isoquercetin, naringenin, protocatechuic, chlorogenic acid, ferulic acid, and myricetin.

No	Compound	Chemical Structure	SMILE
1	Protocatechuic acid (C_7_H_6_O_4_)	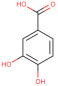	C1=CC(=C(C=C1C(=O)O)O)O
2	Catechin (C_15_H_14_O_6_)	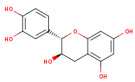	C1[C@H]([C@@H](OC2=CC(=CC(=C21)O)O)C3=CC(=C(C=C3)O)O)O
3	Epicatechin (C_15_H_14_O_6_)	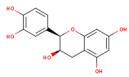	C1[C@H]([C@H](OC2=CC(=CC(=C21)O)O)C3=CC(=C(C=C3)O)O)O
4	Isoquercetin (C_21_H_20_O_12_)	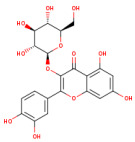	C1=CC(=C(C=C1C2=C(C(=O)C3=C(C=C(C=C3O2)O)O)O[C@H]4[C@@H]([C@H]([C@@H]([C@H](O4)CO)O)O)O)O
5	Naringenin (C_15_H_12_O_5_)	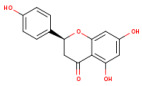	C1[C@H](OC2=CC(=CC(=C2C1=O)O)O)C3=CC=C(C=C3)O
6	Chlorogenic acid (C_16_H_18_O_9_)	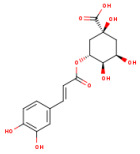	C1[C@H]([C@H]([C@@H](C[C@@]1(C(=O)O)O)OC(=O)/C=C/C2=CC(=C(C=C2)O)O)O)O
7	Ferulic acid (C_10_H_10_O_4_)	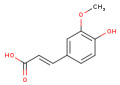	COC1=C(C=CC(=C1)/C=C/C(=O)O)O
8	Myricetin (C_15_H_10_O_8_)	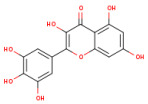	C1[C@H](OC2=CC(=CC(=C2C1=O)O)O)C3=CC=C(C=C3)O
9	Scopolamine (C_17_H_21_NO_4_)	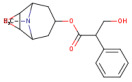	OCC(c1ccccc1)C(=O)OC1CC2N(C(C1)C1C2O1)C
10	Galantamine (C_17_H_21_NO_3_)	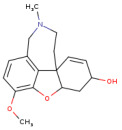	COc1ccc2c3c1OC1C3(CCN(C2)C)C=CC(C1)O

**Table 2:** Phenolic acids and flavonoids identified in *P. americana* extract from raw fruits (PEC) and from dried fruits (PEU).

**Sample**	**Phenolic Compound**	**Concentration** **(µg/mL)**
PEC	Protocatechuic acid	1.89 ± 0.12
	Catechin	2.33 ± 0.48
	Epicatechin	48.96 ± 1.12
	Isoquercetin	10.22 ± 0.99
	Naringenin	21.79 ± 0.15
PEU	Protocatechuic acid	22.93 ± 1.06
	Chlorogenic acid	2.29 ± 0.18
	Ferulic acid	48.29 ± 0.29
	Myricetin	8.07 ± 0.13
	Naringenin	119.07 ± 0.29

**Table 3:** Physicochemical properties of scopolamine (SCO), galantamine (GAL), and polyphenols (epicatechin, naringenin, isoquercetin, catechin, protocatechuic acid, ferulic acid, myricetin, and chlorogenic acid) using Pkcsm and SwissADME platforms.

Descriptor	SCO	GAL	Epicatechin	Naringenin	Isoquercetin	Catechin	Protocatechuic Acid	Acid Ferulic	Myricetin	Chlorogenic Acid
Molecular weight (g/mol)	303.35	287.359	290.27	272.25	464.38	290.27	154.12	194.18	318.24	354.31
Num. heavy atoms	22	21	21	20	33	21	11	14	23	25
Num. arom. heavy atoms	6	6	12	12	16	12	6	6	16	6
Fraction Csp^3^	0.59	0.53	0.20	0.13	0.29	0.20	0.00	0.10	0.00	0.38
Number of rotatable bonds	5	1	1	1	4	1	1	3	1	5
Num. H-bond acceptors	5	4	6	5	12	6	4	4	8	9
Number of H-bonds donors	1	1	5	3	8	5	3	2	6	6
Molar Refractivity	83.48	84.05	74.33	71.57	110.16	74.33	37.45	66.76	80.06	83.50
TPSA (Å)	62.30	41.93	110.38	86.99	210.51	110.38	77.76	12.53	151.59	164.75

**Table 4:** The predicted pharmacokinetic parameters related to drug-likeness and medicinal chemistry of the compounds examined in this study.

Druglikeness	SCO	GAL	Epicatechin	Naringenin	Isoquercetin	Catechin	Protocatechuic acid	Acid ferulic	Myricetin	Chlorogenic Acid
Lipinski	Yes; 0 violation	Yes; 0 violation	Yes; 0 violation	Yes; 0 violation	No; 2 violations: NorO > 10, NHorOH > 5	Yes; 0 violation	Yes; 0 violation	Yes; 0 violation	Yes; 1 violation: NHorOH > 5	Yes; 1 violation: NHorOH > 5
Ghose	Yes	Yes	Yes	Yes	No; 1 violation: WLOGP < −0.4	Yes	No; 3 violations: MW < 160, MR < 40, #atoms < 20	Yes	Yes	No; 1 violation: WLOGP < −0.4
Veber	Yes	Yes	Yes	Yes	No; 1 violation: TPSA > 140	Yes	Yes	Yes	No; 1 violation: TPSA > 140	No; 1 violation: TPSA > 140
Egan	Yes	Yes	Yes	Yes	No; 1 violation: TPSA > 131.6	Yes	Yes	Yes	No; 1 violation: TPSA > 131.6	No; 1 violation: TPSA > 131.6
Muegge	Yes	Yes	Yes	Yes	No; 3 violations: TPSA > 150, H-acc > 10, H-don > 5	Yes	No; 1 violation: MW < 200	No; 1 violation: MW < 200	No; 2 violations: TPSA > 150, H-don > 5	No; 2 violations: TPSA > 150, H-don > 5
Bioavailability Score	0.55	0.55	0.55	0.55	0.17	0.55	0.56	0.85	0.55	0.11

**Table 5:** ADMET prediction of SCO, GAL, and PEC and PEU biocompounds.

Property	Compound Model Name	SCO	GAL	Epicatechin	Naringenin	Isoquercetin	Catechin	Protocatechuic Acid	Acid Ferulic	Myricetin	Chlorogenic Acid	Unit
Absorption	Intestinal absorption (human) (low < 30%, high > 30%)	72.626	94.994	68.829	91.31	47.999	68.829	71.174	93.685	65.93	36.377	Numeric (% Absorbed)
	Skin Permeability (low logKp > −2.5, high logKp < −2.5)	−4.097	−3.75	−2.735	−2.742	−2.735	−2.735	−2.727	−2.72	−2.735	−2.735	Numeric (log Kp)
	P-glycoprotein substrate	Yes	No	Yes	Yes	Yes	Yes	No	No	Yes	Yes	Categorical (Yes/No)
	P-glycoprotein I inhibitor	No	No	No	No	No	No	No	No	No	No	Categorical (Yes/No)
	P-glycoprotein II inhibitor	No	No	No	No	No	No	No	No	No	No	Categorical (Yes/No)
Distribution	VDss (human) (low log VDss < −0.15, high VDss > 0.45)	0.583	0.89	1.027	−0.015	1.846	1.027	−1.298	−1.367	1.317	0.581	Numeric (log L/kg)
	Unbound fraction (human)	0.414	0.36	0.235	0.064	0.228	0.235	0.648	0.343	0.238	0.658	Numeric (Fu)
	BBB permeability (log BB > 0.3 cross BB, log BB < 0.1 do not cross BB)	−0.043	0.081	−1.054	−0.578	−1.688	−1.054	−0.683	−0.239	−1.493	−1.407	Numeric (BB log)
	CNS permeability (log PS > −2 penetrates CNS, log PS < −3 does not penetrate)	−3.031	−2.511	−3.298	−2.215	−4.093	−3.298	−3.305	−2.612	−3.709	−3.856	Numeric (PS log)
Metabolism	CYP3A4 substrate	Yes	Yes	No	No	No	No	No	No	No	No	Categorical (Yes/No)
	CYP1A2 inhibitor	No	No	No	Yes	No	No	No	No	Yes	No	Categorical (Yes/No)
Excretion	Total Clearance	1.096	0.991	0.183	0.06	0.394	0.183	0.551	0.623	0.422	0.307	Numeric (log ml/min/kg)
	Renal OCT2 substrate	No	Yes	No	No	No	No	No	No	No	No	Categorical (Yes/No)
Toxicity	hERG Blockers	0.19	0.458	0.108	0.107	0.013	0.095	0.037	0.036	0.04	0.025	Numeric
	hERG Blockers (10 um)	0.418	0.625	0.677	0.52	0.198	0.694	0.201	0.065	0.706	0.093	Numeric
	DILI	0.104	0.215	0.372	0.22	0.913	0.273	0.573	0.636	0.839	0.291	Numeric
	AMES Toxicity	0.158	0.559	0.604	0.703	0.856	0.618	0.371	0.252	0.657	0.386	Numeric
	Carcinogenicity	0.015	0.726	0.216	0.591	0.239	0.313	0.334	0.249	0.502	0.225	Numeric
	Human Hepatotoxicity	0.895	0.795	0.611	0.673	0.563	0.691	0.396	0.702	0.325	0.543	Numeric
	Drug-induced Nephrotoxicity	0.403	0.802	0.083	0.328	0.064	0.173	0.13	0.407	0.003	0.441	Numeric
	Drug-induced Neurotoxicity	0.753	0.744	0.101	0.644	0.002	0.201	0.076	0.212	0.001	0.009	Numeric
	Hematotoxicity	0.16	0.505	0.133	0.075	0.038	0.067	0.156	0.177	0.015	0.028	Numeric
	Genotoxicity	0.677	0.739	0.975	0.978	0.976	0.956	0.445	0.12	0.995	0.243	Numeric
	RPMI-8226 Immunotoxicity	0.043	0.097	0.035	0.099	0.031	0.042	0.007	0.034	0.007	0.016	Numeric
	A549 Cytotoxicity	0.058	0.069	0.959	0.421	0.698	0.937	0.129	0.018	0.938	0.203	Numeric

The intestinal environment provides several key features that influence drugs.

## Data Availability

The data presented in this study are available on request from the corresponding author.
